# How Entorhinal Grid Cells May Learn Multiple Spatial Scales from a Dorsoventral Gradient of Cell Response Rates in a Self-organizing Map

**DOI:** 10.1371/journal.pcbi.1002648

**Published:** 2012-10-04

**Authors:** Stephen Grossberg, Praveen K. Pilly

**Affiliations:** Center for Adaptive Systems, Graduate Program in Cognitive and Neural Systems, and Center for Computational Neuroscience and Neural Technology, Boston University, Boston, Massachusetts, United States of America; Indiana University, United States of America

## Abstract

Place cells in the hippocampus of higher mammals are critical for spatial navigation. Recent modeling clarifies how this may be achieved by how grid cells in the medial entorhinal cortex (MEC) input to place cells. Grid cells exhibit hexagonal grid firing patterns across space in multiple spatial scales along the MEC dorsoventral axis. Signals from grid cells of multiple scales combine adaptively to activate place cells that represent much larger spaces than grid cells. But how do grid cells learn to fire at multiple positions that form a hexagonal grid, and with spatial scales that increase along the dorsoventral axis? *In vitro* recordings of medial entorhinal layer II stellate cells have revealed subthreshold membrane potential oscillations (MPOs) whose temporal periods, and time constants of excitatory postsynaptic potentials (EPSPs), both increase along this axis. Slower (faster) subthreshold MPOs and slower (faster) EPSPs correlate with larger (smaller) grid spacings and field widths. A self-organizing map neural model explains how the anatomical gradient of grid spatial scales can be learned by cells that respond more slowly along the gradient to their inputs from stripe cells of multiple scales, which perform linear velocity path integration. The model cells also exhibit MPO frequencies that covary with their response rates. The gradient in intrinsic rhythmicity is thus not compelling evidence for oscillatory interference as a mechanism of grid cell firing. A response rate gradient combined with input stripe cells that have normalized receptive fields can reproduce all known spatial and temporal properties of grid cells along the MEC dorsoventral axis. This spatial gradient mechanism is homologous to a gradient mechanism for temporal learning in the lateral entorhinal cortex and its hippocampal projections. Spatial and temporal representations may hereby arise from homologous mechanisms, thereby embodying a mechanistic “neural relativity” that may clarify how episodic memories are learned.

## Author Summary

Spatial navigation is a critical competence of all higher mammals, and place cells in the hippocampus represent the large spaces in which they navigate. Recent modeling clarifies how this may occur via interactions between grid cells in the medial entorhinal cortex (MEC) and place cells. Grid cells exhibit hexagonal grid firing patterns across space and come in multiple spatial scales that increase along the dorsoventral axis of MEC. Signals from multiple scales of grid cells combine to activate place cells that represent much larger spaces than grid cells. This article shows how a gradient of cell response rates along the dorsoventral axis enables the learning of grid cells with the observed gradient of spatial scales as an animal navigates realistic trajectories. The observed gradient of grid cell membrane potential oscillation frequencies is shown to be a direct result of the gradient of response rates. This gradient mechanism for spatial learning is homologous to a gradient mechanism for temporal learning in the lateral entorhinal cortex and its hippocampal projections, thereby clarifying why both spatial and temporal representations are found in the entorhinal-hippocampal system.

## Introduction

### A gradient of spatial scales in medial entorhinal cortex

Navigating the world requires the brain to learn and maintain memory of spatial positions within various environments. Place cells in the hippocampal areas CA1 and CA3 demonstrate a neural code for position in large spaces that higher mammals inhabit [1] and thereby play a critical role in spatial navigation. CA3 receives major projections from layer II of the medial entorhinal cortex (MEC) [2], where grid cells are predominant [3], [4]. Unlike place cells, individual grid cells fire at multiple positions. When an animal navigates in an open field, these positions form a regular hexagonal grid uniformly covering the entire navigable environment. These cells are found throughout the length of MEC with the spatial period of their firing fields increasing from the dorsomedial to the ventrolateral end [4]–[6].

In particular, Brun and colleagues [6] recorded from a total of 143 grid cells within layers (II, III, V/VI) of MEC located between 1% to 75% the distance along the dorsoventral axis, while rats ran back and forth on a 18 m long linear track. The recorded cells were divided into three groups based on their anatomical location with respect to the postrhinal border of MEC; namely, dorsal, intermediate and ventral. The one-dimensional periodic spatial responses of these cells in the two running directions were processed separately to estimate characteristic properties of grid cells, such as grid spacing, grid field width, peak firing rate, and mean firing rate. The main finding was that both grid spacing and field width increased from dorsal group to ventral group, for either running direction. Interestingly, distributions of these variables increased not only in mean but also in variability with distance along the dorsoventral axis. However, the peak firing rate decreased from dorsal group to ventral group, and there was a negative trend for mean firing rate.

The presence of multiple spatial scales on the dorsoventral axis of MEC has important implications for the development of the hippocampal place cells [7]–[9]. Several self-organizing map (SOM) models have been proposed that show how signals from grid cells of multiple spatial scales can together induce the learning of hippocampal place cells that are capable of representing position in the larger spaces that higher mammals navigate (e.g., [10], [11]). In particular, Gorchetchnikov and Grossberg [11] showed this expansion of the scale of the spatial representation from grid cells to place cells arises due to the fact that the SOM is sensitive to the most frequent coactivations of grid cells across multiple scales, which on a linear track occur with a spatial period equal to the least common multiple of the inducing grid spacings. But how do grid cells learn to fire at multiple positions that form a hexagonal grid in two-dimensional open environments? And how does the spatial scale of grid cells increase along the dorsoventral axis of MEC, enabling their target place cells to represent ever-larger spaces? Recent data and modeling provide some clues, forming the basis for the current work.

### Correlating stellate cell oscillation frequency with grid cell spatial scale

Excitatory projections to the hippocampal formation from layer II of MEC are primarily from stellate cells [12]. That makes them the most likely candidates for grid cells. *In vitro* whole-cell patch clamp recordings [13], [14] have shown that these stellate cells exhibit subthreshold membrane potential oscillations (MPOs) in response to steady current injection. The temporal period of these oscillations increases from the dorsomedial to the ventrolateral end of MEC, thereby correlating with the observed gradient in spatial period and size of the firing fields of grid cells. In addition, voltage-clamp recordings in these cells demonstrated that the time constants of the hyperpolarization-activated cation current 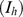 decreases along the dorsoventral axis of MEC [15], [16]. Knockout of the HCN1 subunit in the hyperpolarization-activated cyclic nucleotide-gated (HCN) channels, which control 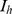 kinetics [17], flattens the dorsoventral gradient of MPO frequency [18]. In addition, the rise and fall times of excitatory postsynaptic potentials (EPSPs) in these cells progressively become longer along the dorsoventral axis [19]. The variation in EPSP kinetics was attributed to differences in the membrane conductance mediated by HCN and leak potassium channels. Combined, all these results suggest a correlation between the rate of intrinsic dynamics in MEC layer II stellate cells and the spatial scale of grid cells.

### Model accomplishments

This article develops a SOM neural model, called Spectral Spacing for reasons summarized below, to explain the above data. This model shows how a gradient of cell response rates along the dorsoventral axis of MEC can control the development of grid cells whose hexagonal grid firing fields exhibit a gradient of spatial scales and whose MPOs exhibit a gradient of frequencies. These results combine several conceptual and technical advances.

First, these results are part of an emerging general entorhinal-hippocampal model architecture (see also [20]), which shows that, despite their different receptive field structures, both grid cells and place cells may be learned using the same SOM laws. Thus, both grid cell periodic hexagonal firing fields and place cell unimodal firing fields, despite their different appearances, may arise from the same neural mechanisms due to the different inputs that they receive at their respective stages in the entorhinal-hippocampal hierarchy.

Second, these SOM laws have been proposed to control development and learning in many other parts of the brain, notably the visual cortex. Thus, both grid and place cells may develop using general SOM principles of brain map organization.

Third, the linear velocity and angular velocity path integration inputs that drive model learning are derived from realistic trajectories of rats in spatial learning and memory experiments.

Fourth, these linear velocity and angular velocity estimates can both be transformed into position codes by ring attractors.

Fifth, the rate gradient mechanism for spatial learning in the MEC pathway and its hippocampal projections is homologous to a rate gradient mechanism that has been used to model temporal learning in the lateral entorhinal cortex (LEC) pathway and its hippocampal projections. Spatial and temporal representations in the medial and lateral processing streams may hereby arise from homologous mechanisms, thereby embodying a mechanistic “neural relativity” in the entorhinal-hippocampal system. This homology may clarify why spatial and temporal representations both occur in hippocampus, and provides new clues about how episodic memories may be learned.

In summary, this model system exhibits parsimony and unity, both in its use of similar ring attractor mechanisms to code the linear and angular velocity path integration inputs that drive learning, and in its use of a rate gradient mechanism that can support the learning of both spatial and temporal codes.

Even more striking is the fact that both grid cell and place cell receptive fields emerge by detecting, learning, and remembering the most frequent and energetic coactivations of their inputs. This co-occurrence property is different from the property of oscillatory interference that some other models have proposed (e.g., [21]). Oscillatory interference models have, to the present, been used to explain properties of grid cells, without showing how they can be learned, or how such a learning process can generate the different grid spatial scales along the dorsoventral extent of MEC. Moreover, several articles (e.g., [13], [22]) have interpreted the gradient of subthreshold MPO frequencies in MEC layer II stellate cells as strong evidence for an oscillatory interference-based firing of grid cells. In sharp contrast, the grid cells in the Spectral Spacing model exhibit the gradient of MPO frequencies as an epiphenomenon of SOM learning mechanisms, thereby showing that this gradient can occur in the absence of an oscillatory interference mechanism.

In order to better understand what aspects of the Spectral Spacing model are needed to explain how spatial and temporal properties of grid cell firing change along the dorsoventral extent of MEC, several model and input variations were simulated (see **Simulation Settings**). These simulations demonstrate that, at least among these variations, only a response rate gradient, combined with input cells that have normalized receptive fields, can explain all the data mentioned above.

### Stripe cells and ring attractors

**Figure 1:**
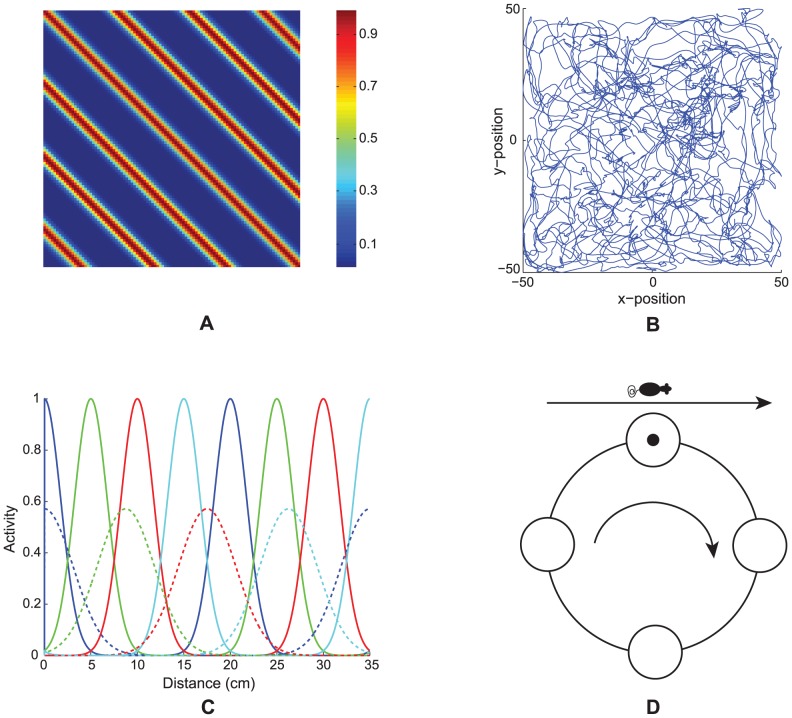
Linear path integration inputs. (A) Spatial response of a model stripe cell with a spacing of 20 cm in a 100 cm×100 cm environment. (B) Realistic rat trajectory in the same sized environment used in our simulations (data: [4]). (C) Small-scale (solid; spacing of 20 cm) and large-scale (dashed; spacing of 35 cm) stripe fields of four spatial phases (colors) along their preferred direction. Note the normalized stripe fields; that is, the area under each stripe field is a constant between the two scales. (D) Depiction of how the bump of activity in each directional ring attractor can be moved by linear movements of an animal with a component along the preferred direction.

The input cells to the grid cells are called *stripe cells* [23]. They are called stripe cells because each cell fires with a preferred movement direction and spatial period, thereby giving rise to stripes of activation (Figure 1A). Suggestive data about these cells in deeper layers of MEC were reported in [4]. In addition, Krupic, Burgess, and O'Keefe [24] have reported data showing stripe-like spatial firing profiles for a group of cells in the dorsal parasubiculum, which projects to layer II of MEC [25], [26].

In the GRIDSmap model [23] and the Spectral Spacing model simulations, the stripe cells process linear velocity inputs that are modulated by head direction as the model animal navigates a realistic trajectory that was reported in the data of [4]; see Figure 1B. These signals are assumed to be computed *in vivo* from vestibular estimates of linear and angular acceleration, which are generated in the otolithic organs and semicircular canals, respectively, of the inner ears [27].

In addition to its preferred direction and spatial scale, each stripe cell is assumed to have a preferred spatial phase (Figure 1C). A set of stripe cells for a given direction and spacing, which differ only in spatial phase, can be represented by cells constituting a one-dimensional ring attractor (Figure 1D). In such a ring attractor, linear velocity projected onto the preferred direction moves an activity bump around the ring of stripe cells (see Figure 1D and Equations 1.1 1.4). One revolution of the activity bump corresponds to traversal of a length equal to the associated stripe spacing along the direction (Figure 1A). The spatial firing of a stripe cell as the animal moves at a constant speed on a straight path is assumed to have a Gaussian profile, for simplicity, with different stripe cells in the ring having different spatial offsets for their peak firing. The movement of the activity bump depends on the component of linear velocity along the associated direction. As a result, the spatial firing pattern of a given stripe cell in a two-dimensional environment resembles Gaussian-modulated oriented stripes with a fixed spacing that uniformly spread across the entire environment (Figure 1A). Because of the periodic boundary condition, each stripe cell operates over a limited spatial scale equivalent to the spacing between its adjacent stripe fields.

As noted above, each stripe cell ring attractor includes cells that are sensitive to a given spatial scale, both spatial period and spatial phase, and movement direction. The set of all stripe cells, across all spatial periods, spatial phases, and directions, taken together, implicitly represent the spatial position of the animal. In particular, stripe cells of different spacings can represent the animal's position at multiple spatial resolutions.

### Head direction cells and ring attractors

The firing of a stripe cell with a prescribed directional preference is modulated by a head direction signal via a cosine law that projects the current direction of the navigating animal at each time onto the stripe cell's preferred direction (see Equation 1.1). Head direction estimates have been modeled by ring attractors that are sensitive to angular velocity signals [28]–[35]. Both linear velocity and angular velocity signals in the Spectral Spacing model are thus assumed to be transformed into movements of activity bumps in ring attractors in order to perform linear and angular path integration, respectively (cf. [23], [36]). Adult-like head direction cells are already present in the parahippocampus by P16 when rat pups begin to explore their environments for the first time [37], [38]. If both stripe cells and head direction cells are indeed computed by ring attractors, then this provides a plausible explanation of how stripe cells could be ready at this developmental stage to support the learning of grid cells.

### SOM dynamics and learning

Stripe cells with multiple directional preferences and spatial phases for a given spatial period initially project with random adaptive weights to cells in the category learning layer of a SOM. SOM cells obey membrane, or shunting, equations and interact in a recurrent on-center off-surround network. Self-excitatory feedback enables the resolution of competition among the map cells in order to choose one or a few winners. The self-excitatory feedback does this by contrast-enhancing the activity of winning category cells [39], but it can also cause perseveration of activity in the winning cells, even after their bottom-up inputs shut off. A perseverating cell could inhibit other map cells, via the recurrent off-surround, that would be needed to represent different combinations of inputs that arise as an animal continues to navigate. Activity-dependent habituative gating of the positive feedback signals causes a collapse of such persistent self-activation, and thereby allows different map cells to become active and learn at different times as the bottom-up stripe cell input pattern changes with the animal's navigational movements in space. In other words, habituative gating helps to “whiten” the learned spatial fields of the map cells. Habituative gating has been used in SOM models of other parts of the brain since being introduced in [40]. It has helped, for example, to simulate complex properties of map development in visual cortical area V1 (e.g., [41]–[43]).

Signals from winning map cells trigger learning in the abutting synapses of pathways from the stripe cells. The adaptive weights in these synapses track a normalized time-average of the signals in the pathways from the stripe cells while their target map cells are active. After learning, the bottom-up signals can efficiently activate map cells that exhibit hexagonal grid fields.

In addition to these basic SOM ingredients, the current model investigates how a gradient of response rates in the map cells can lead to learning of a gradient of model grid cell spatial scales whose properties match neurophysiological data from multiple experiments about grid cells along the dorsoventral axis of the MEC. See the subsection below on the **Scale selection problem**.

The learning law is called a *competitive instar learning law* because it selectively strengthens the adaptive weights from coactive stripe cells to active map cells while it competitively self-normalizes the total adaptive weight abutting each map cell [40], [41], [44], [45]. This learning law enables each grid cell to arise as a learned spatial category in a SOM. The competitive aspect in the learning law may be interpreted in terms of how developing axons abutting a target neuron compete for limited target-derived neurotrophic factor support in order to survive [46]–[48], and its conservation of total synaptic weight is consistent with neurobiological data (e.g., [49]).

Such a competitive instar learning law is different from a purely Hebbian learning law, which allows adaptive weights to increase but does not allow them to decrease. The instar learning law permits both weight increases (long-term potentiation) and weight decreases (long-term depression). It hereby enables the weights to adapt to the *spatial pattern* of signals from the stripe cells. This pattern sensitivity enables grid cell learning to become sensitive to temporal co-occurrences of stripe cell firing.

Simultaneously active stripe cells are more likely to strongly activate map cells whose bottom-up weight patterns closely match their activity pattern. Adaptation of the weights to a map cell occurs only when its activity is above a threshold (see 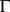 in Equation 1.6). This postsynaptic activity-based gating ensures faster adaptation of incoming weights for more active map cells. During each learning episode, the weights tend towards the average normalized pattern of the inputs. Thus, the likelihood of the map cells becoming tuned to particular sets of inputs, which consistently succeed in driving them, gradually increases. Note that the bottom-up connections from stripe cells to grid cells remain adaptive for the lifetime of the animal, and not just during the development period.

The GRIDSmap model [23] learned grid cells in response to a wide choice of stripe cell directional preferences. For example, hexagonal grid firing fields were learned even when stripe cell directions differed by 7, 10, 15, 20, 60, or random numbers of degrees. GRIDSmap hereby overcame a problem of the oscillatory interference models of grid cells (e.g., [21], [22]), which created a hexagonal grid spatial firing pattern using hard-wired inputs from exactly three band cells (a similar concept to stripe cells, proposed earlier by [21]) with directional preferences differing by 60°. Band cells in oscillatory interference models, unlike stripe cells, are defined by the interference of two theta frequency MPOs. SOM models are, in contrast, able to select among multiple possible combinations of stripe cell inputs to learn only a subset of combinations that are favored in terms of both frequency and total activation. Why hexagonal grid patterns are favored can be explained in terms of a simple trigonometric property of two-dimensional space to which a SOM is sensitive as an animal navigates [20], [23]. By this property, among all possible subsets of coactive stripe cells experienced during two-dimensional navigation, the ones that are most frequent and energetic are those comprising three stripe cells whose directional preferences differ from each other by 60° [20], [23]. These favored coactivations of stripe cells occur at positions that form a regular hexagonal grid when the model animal navigates in an open field.

### SOM hierarchy: From stripe cells to place cells via grid cells

Until recently, SOM models of place cell learning used idealized or hand-crafted grid cells (e.g., [10], [11]). Pilly and Grossberg [20] proposed the GridPlaceMap model to show how grid and place receptive fields, despite their different characteristics, can emerge simultaneously at different levels in a SOM hierarchy, obeying the same laws for neuronal dynamics and synaptic plasticity, by responding to the most frequent and energetic coactivations of their corresponding input neurons. This medial entorhinal-hippocampal hierarchy of stripe, grid, and place cells enables the brain to represent increasingly large spaces, and provides increasingly large spatial information per cell in predicting the spatial position of an animal.

### Scale selection problem

**Figure 2:**
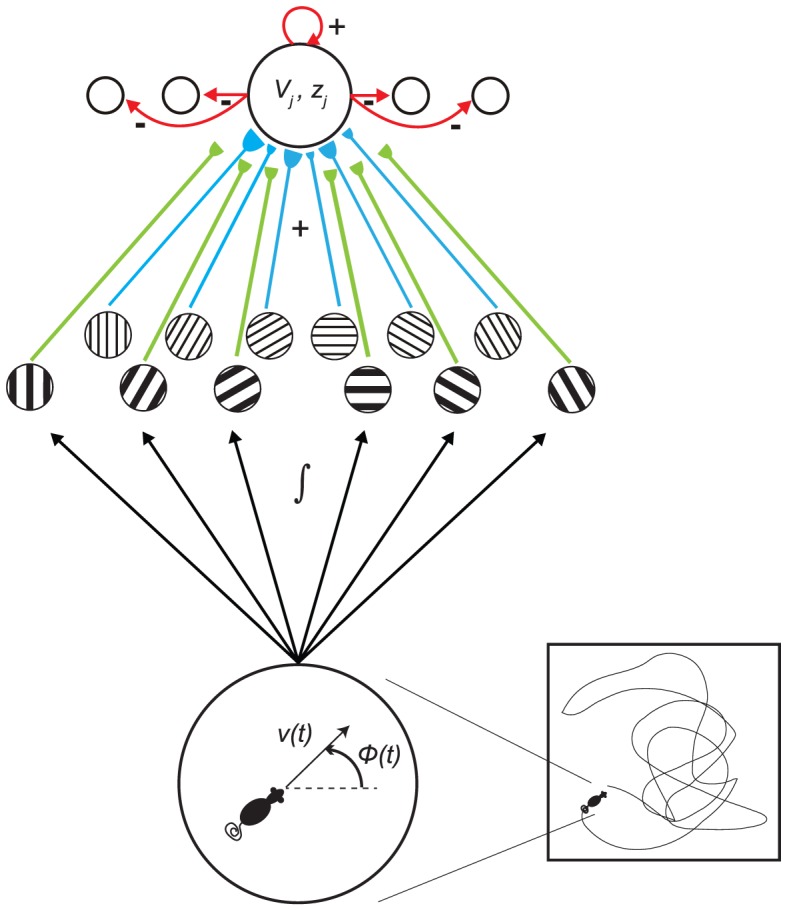
Model depiction. The Spectral Spacing model responds to the navigational movements of an animal along a realistic trajectory and with stripe cells of multiple spatial scales initially projecting to the population of category learning cells at some location along the dorsoventral axis of MEC. Model simulations were conducted with 25 category cells in each of 10 MEC local populations that differed in the rate of intrinsic cellular dynamics, and with input stripe cells of nine directional preferences, four spatial phases, and up to three spatial scales.

Both the GRIDSmap and the GridPlaceMap models learn hexagonal grid firing fields whose spatial scale is derived from that of the input stripe cells. In particular, stripe cells with the same period were used to learn grid fields of a given spatial scale. Stripe cells of different spatial scales were assumed to activate different locations along the dorsoventral axis in layer II of MEC, thereby giving rise to grid cells with different spatial scales. But how is the selection of just one spatial scale of stripe cells realized for each grid cell scale? What would happen if stripe cells of multiple scales initially projected to the map layer before grid cell learning began, as in Figure 2? In other words, how do grid cells learn to select among, not only multiple directional preferences and spatial phases, but also among the multiple spatial scales, of their stripe cell inputs? What properties of the dynamics of a map cell can select the spatial scale to which it will learn to respond as a grid cell?

### Cell response rates select grid cell spatial scale and controls MPO frequency

**Figure 3:**
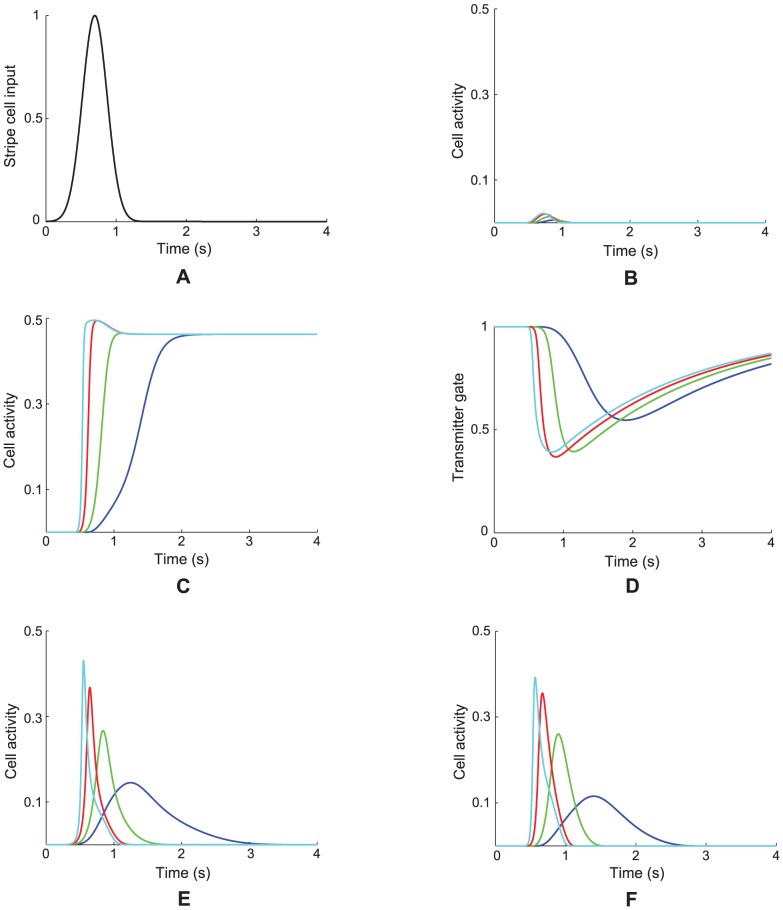
Case 1 results. Results of Case 1 in which a single category cell responded to a stripe cell-like input 
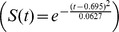
 shown in (A). (B) Cell responses defined by 
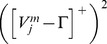
 for different response rates (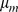: 1 (cyan), 0.5 (red), 0.2 (green), and 0.1 (blue)) in the absence of self-excitatory feedback. Here, cell potential 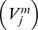 follows: 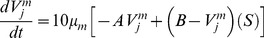. (C) Cell responses defined by 
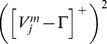
 in the presence of self-excitatory feedback that is not habituatively gated. In this case, cell potential 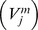 follows: 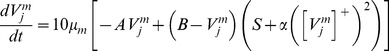. (D) Dynamics of the habituative transmitter 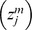 in the presence of self-excitatory feedback that is habituatively gated. (E) Cell responses defined by the habituatively gated product 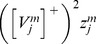 for the case in (D). (F) Cell responses defined by 
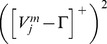
 for the case in (D). For (D–F), cell potential 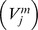 and habituative transmitter 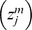 follow 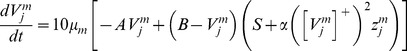 and 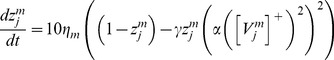, respectively.

This article shows that the rates at which the category cells and their corresponding habituative transmitters respond, called the response rate (parameter 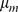 in Equation 1.5) and habituation rate (parameter 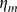 in Equation 1.7), respectively, can help to select the spatial scale of the stripe cells to which the category cells will learn to respond, and thus the spatial scale of the learned hexagonal grid firing fields, as well as the MPO frequencies with which these grid cells respond *in vitro* to a steady current input. Whereas a dorsoventral gradient in either response rate or habituation rate can explain the corresponding gradient in learned spatial scale and MPO frequency of grid cells, only a gradient in response rate was found to be consistent with data regarding the associated dorsoventral gradient in peak and mean firing rates of grid cells [6]; see the **Results** section for details. Different cell response rates also indirectly alter the rates at which the habituative transmitters inactivate and recover (see Figure 3D).

### Spectral Timing and Spectral Spacing

Remarkably, this response rate gradient for *spatial* learning is computationally homologous to a rate gradient that was proposed over 20 years ago to explain hippocampal data about *temporal* learning [50]–[52]. The model for temporal learning was called a Spectral Timing model because its different cell populations respond with a “spectrum” of different rates. The current model may therefore be called a Spectral Spacing model. Whereas the rate gradient for spatial learning is proposed to occur in MEC and its hippocampal projections, the rate gradient for temporal learning is proposed to occur in LEC and its hippocampal projections. This homology may provide new clues about how episodic memories are learned. See the **Discussion** section for further comments about this predicted form of “neural relativity” in the entorhinal-hippocampal system.

## Methods

The Spectral Spacing model that is developed in this article significantly refines and modifies the GRIDSmap model of [23] to explain how a cell response rate gradient [19] can generate learning of a gradient in grid cell spatial scale [5], [6] from among multiple spatial scales of input stripe cells. In addition, the learned grid cells exhibit activity patterns whose properties simulate data about the gradient of MPO frequency [13], [14] and of peak and mean firing rates [6] along the dorsoventral axis in layer II of MEC. The Spectral Spacing model also computationally investigates different variations of stripe cell properties (peak firing rate, stripe field width) across spatial scales to predict what may be observed in future experiments. Besides these major conceptual advances, the Spectral Spacing model also incorporates several technical advances over the GRIDSmap model that enable it to learn a greater number of stable grid cells in a larger population of self-organizing cells; see the **Differences with GRIDSmap model** subsection in the **Discussion** section.

We first provide below a complete mathematical description of the Spectral Spacing model and its variations. The values of parameters that do not differ across simulation cases are listed in Table 1. The values for the other parameters are specified in the **Simulation Settings** subsection below. Table 2 lists experimental evidence in support of the various model components. Numerical integration was performed using Euler's forward method with a fixed time step 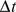.

**Table 1:** Values of model parameters that do not differ across various simulation cases.

A	B	C	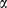	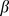	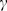	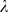	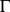	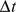 (s)
3	1	0.5	17.5	1.5	0.2	0.025	0.1	0.002

**Table 2:** List of experimental evidence for various model components.

*Model component*	*Equation reference*	*Experimental evidence*
Stripe cells in parasubiculum (preliminary SfN abstract)	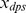 in Equation 1.5	[24]
Anatomical projections from parasubiculum to layer II of MEC	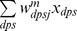 in Equation 1.5	[25], [26]
Dorsoventral gradient in the rate of temporal summation of MEC layer II stellate cells	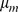 in Equation 1.5	[19]
Self-excitatory feedback in MEC layer II stellate cells based on a Ca^2+^-sensitive nonspecific cation current (*I_CAN_*)	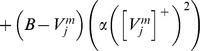 in Equation 1.5	[53], [54]
Inhibitory interneurons in layer II of MEC	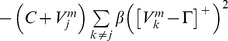 in Equation 1.5	[55]
Adaptation in MEC layer II stellate cells related to Ca^2+^-dependent K^+^ (AHP) currents	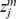 in Equation 1.5; and Equation 1.7	[56]
Competition among developing axons abutting a map cell	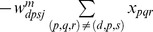 in Equation 1.6	[47], [48]
Conservation of total synaptic weight	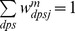 when Equation 1.6 converges	[49]
Postsynaptic activity-dependent plasticity	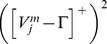 in Equation 1.6	[57]

### Spectral Spacing model equations

#### Stripe cells

As noted above, stripe cells integrate linear velocity in multiple directions, spatial phases, and spatial scales in ring attractor circuits. They are algorithmically computed, for simplicity, as follows [23]: If at time 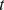 an animal heads along allocentric direction 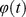 with velocity 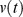, then the velocity 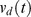 along direction 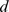 is:
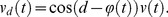
(1.1)The displacement 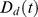 traversed along direction 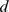 with respect to the initial position is calculated by path integration of the corresponding velocity:
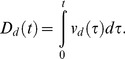
(1.2)This directional displacement variable is converted into activations of stripe cells that prefer different spatial phases 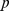 along a ring attractor that is selectively tuned to direction 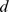 and spatial scale 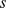. Let 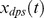 be the activity of a stripe cell whose spatial fields are oriented perpendicular to direction 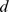 with spatial phase 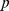 and spatial period 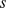. This stripe cell has maximal activity at periodic positions 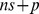 along direction 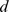, for all integer values of 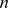; see Figure 1A. Activity 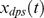 will thus be maximal whenever (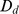 modulo 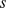) = 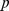, where the modulo operator computes the remainder when 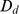 is divided by 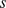, and thus resets the displacement modulo the period 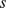. This periodically reset displacement, computed with respect to spatial phase 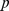 is:
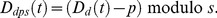
(1.3)Thus, if the stripe cell 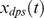 has a Gaussian-like spatial firing profile, then its activity can be modeled as:
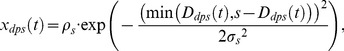
(1.4)where 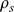 is the maximal activity and 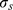 is the standard deviation of each of its individual stripe fields along the direction 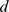. The simulations were carried out with two, or three, spatial scales 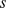 of stripe cells converging on individual category cells. Learning determines which stripe cell spatial scale gains control of each category cell through time, and how that results in its learned grid scale. Simulations demonstrate how the response rate of a category cell determines its learned grid scale. The directional displacement variables 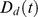 were all initialized to 0 at the start of each learning trial.

#### Category cells

The membrane potential 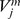 of the MEC layer II category cell 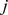 in population 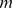 along the dorsoventral axis obeys shunting dynamics within a recurrent on-center off-surround network [40], [44]. The membrane potential 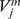 of the 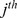 cell in population 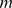 therefore obeys the equation:
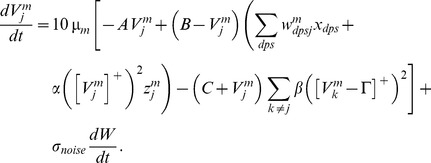
(1.5)The model was run in several variations to demonstrate the effects of gradients in cell response rates or habituation rates. This analysis points to the fact that a gradient of response rates 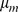, with all other parameters held fixed, leads to learned grid cells that best match neurophysiological data. Thus, in one set of simulations, 10 non-interacting populations of category cells, each with 25 cells, were assumed to occur at different anatomical locations on the dorsoventral axis. The only parameter that was varied across these populations was the response rate 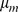, with values of 1, 0.9, 0.8, 0.7, 0.6, 0.5, 0.4, 0.3, 0.2, and 0.1. This is similar to the anatomical gradient of response rates proposed in the Spectral Timing model to account for the learning of adaptively timed behaviors [50].

In Equation 1.5, 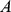 is the decay parameter corresponding to the leak conductance; 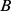 and 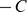 are the reversal potentials of the excitatory and inhibitory channels, respectively; 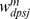 is the synaptic weight of the projection from the stripe cell with activity 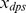 in Equation 1.4 to the category cell 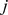 in population 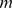; 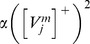 is the on-center self-excitatory feedback signal of the cell, which helps to resolve the competition among category cells within cell population 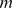, where 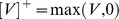 defines a threshold-linear function, and 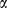 is the gain coefficient; 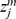 is the habituative transmitter gate of category cell 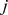; 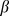 is the connection strength of the inhibitory signal 
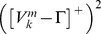
 from category cell 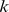 in the off-surround to category cell 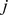 within population 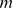; and term 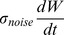 injects additive noise into the cellular dynamics, where 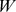 is a Brownian motion process with independent increments sampled from a Gaussian distribution with zero mean and standard deviation equal to 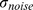. At each time step (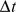) of numerical integration, a zero mean Gaussian random variable of variance 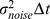 is added to the cell potential. The output activity of category cell 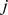 is given by 
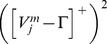
, which is the same as its recurrent inhibitory signal to other cells in the population. The membrane potential of each category cell was initialized to 0 at the start of each learning trial.

#### Adaptive weights

The adaptive weights 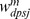 of projections from stripe cells to category cells are governed by a variant of the competitive instar learning law [40], [41]:
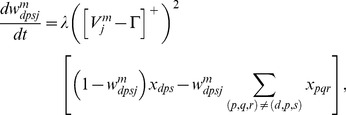
(1.6)where 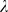 is the learning rate; the category cell output signal 
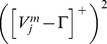
 gates learning on and off; and the learning rule defines a self-normalizing competition among afferent synaptic weights to the target cell, leading to a maximum learned total weight to the cell of 1. Each weight 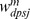 was initialized to a random value drawn from a uniform distribution between 0 and 0.1 at the start of the first learning trial.

Equation (1.6) can be rewritten with term 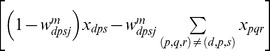 replaced by 
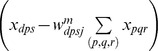
, which shows that the weight 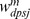 is attracted to a time-average of the ratio of input activities during the times when the gating, or learning, signal 
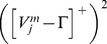
 is positive. This fact embodies the intuition that the learning law conserves the total number of synaptic learning sites at each map cell by a homeostatic combination of excitatory and inhibitory influences.

#### Habituative gating

The habituative transmitter 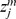 of category cell 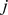 in population 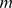 is defined by:
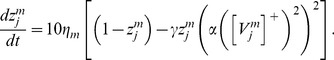
(1.7)In Equation 1.7, 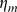 determines the response rate of the transmitter dynamics (called the habituation rate) and 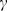 modulates the depletion rate of the transmitter. The habituative transmitter of each category cell was initialized to its maximum value of 1 at the start of each learning trial.

Intuitively, Equation 1.7 says that the transmitter 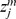 is depleted, or inactivated, via mass action by the signal that it gates (see Equation 1.5). In particular, term 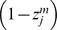 controls the gate recovery rate to the target level of 1, and term 
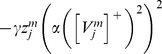
 controls the gate inactivation rate, which is proportional to the current gate strength 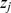 times the square of the signal 
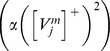
 that 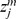 gates in Equation 1.5. The squaring operation causes the gated signal to first increase and then decrease through time in response to excitatory input (cf., [58]), thereby limiting the duration of intense cell activity, and thus cell perseveration. This duration is inversely proportional to both the response rate 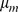 (see Figure 3F) and the habituation rate 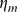.

### Post-processing

The 100 cm×100 cm environment was divided into 2.5 cm×2.5 cm bins. During each learning trial, the amount of time spent by the navigated trajectory in the various spatial bins was tracked. The output activity of each category cell in every spatial bin was accumulated as the trajectory visited that bin. The occupancy and activity maps were smoothed using a 5×5 Gaussian kernel with standard deviation equal to one. At the end of each learning trial, smoothed and unsmoothed rate maps for each category cell were obtained by dividing the cumulative activity variable by cumulative occupancy variable in each bin. Peak and mean firing rates for a category cell in a given trial were obtained by considering all spatial bins in the corresponding rate map. For each category cell, six local maxima with 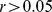 and closest to the central peak in the spatial autocorrelogram of its smoothed rate map were identified. Gridness score, related to rotational symmetry, was then derived using the method described in [38], and grid spacing was defined as the median of the distances of these six local maxima from the central peak [5]. Grid orientation was defined as the smallest positive angle with the horizontal axis made by line segments connecting the central peak to each of these local maxima [5]. Grid field width was estimated by computing the width of the central peak in the spatial autocorrelogram at which the correlation equals zero or there is a local minimum, whichever is closer to the central peak [37]. Further, inter-trial stability of each category cell for a given trial was computed as the correlation coefficient between its smoothed rate maps from the current and immediately previous trials, considering only those bins with rate greater than zero in at least one of the two trials [38]. A gridness score greater than 0 was used to classify map cells as having hexagonal grid-like spatial firing fields.

### Current injection paradigm

*In vitro* experiments by [13] and [14] were simulated by injecting steady current input 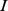 into the category cells in the absence of bottom-up inputs 
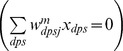
 and local recurrent inhibitory interactions 
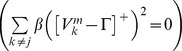
. The membrane potential 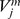 of each category cell in this paradigm was obtained using Equation 1.5:
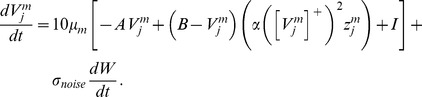
(1.8)The habituative transmitter gate 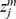 was defined once again by Equation 1.7. The membrane potential trace of each cell for the duration of the current injection was used to estimate the underlying frequency of the MPO as the one maximizing its power spectrum. The power spectrum was calculated using the Fast Fourier Transform (FFT) of the potential trace after subtracting its mean.

### Spectral Spacing model variations

We considered two variations of the model equations to clarify what combination of mechanisms best explains neurobiological data.

#### Variation 1

This variation uses the same habituative gating and learning laws as in the GRIDSmap model [23]:

*Category cells:*

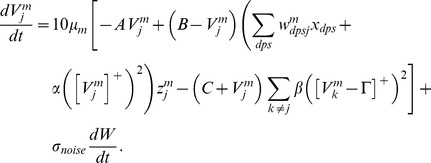
(2.1)


*Adaptive weights:*

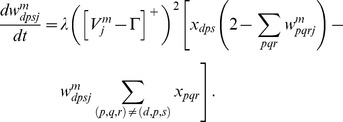
(2.2)


*Habituative gating:*

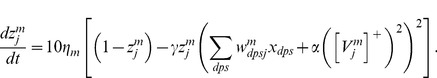
(2.3)In the learning Equation 2.2, all the weights compete for a constant total available weight (chosen to be 2) via term 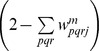, rather than just the weight of the corresponding projection, as in term 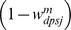 in Equation 1.6. The advantages of the current learning law are summarized below in the subsection that compares the current model properties with those of GRIDSmap.

#### Variation 2

This variation uses the Spectral Spacing equations, with the addition that the recurrent inhibitory feedback and output signals are also habituatively gated; see term 
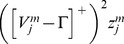
 of Equation 3.1:

*Category cells:*

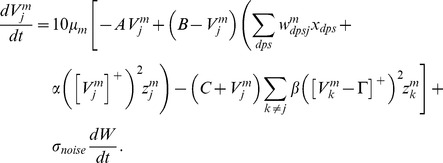
(3.1)


*Adaptive weights:*

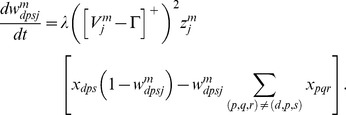
(3.2)


*Habituative gating:*

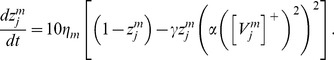
(3.3)


### Simulation settings

Stripe cells were simulated with two, or three, spatial periods (two: 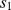 = 20 cm, 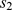 = 35 cm; three: 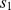 = 20 cm, 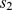 = 35 cm, 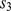 = 50 cm), four spatial phases (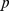 = [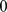, 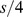, 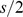, 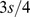] for the stripe period 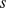), and nine direction preferences (−80° to 80° in steps of 20°). Stripe cells were activated in response to linear velocity and head direction inputs derived from a realistic rat trajectory of ∼10 min in a 100 cm×100 cm environment (data: [4]); see Figure 1B. The trajectory was interpolated to increase its temporal resolution to match with the time step of numerical integration of model dynamics (2 ms), and it was assumed that the head direction was parallel to the trajectory at any moment.

In each of the Cases 2–11 below, 40 learning trials were employed. For these simulations except those in Case 3, the model animal ran along the trajectory shown in Figure 1B in each trial. For Case 3, a novel trajectory was created for each trial by rotating the original trajectory by a random angle about the origin. In order to ensure that such derived trajectories go beyond the square environment only minimally, the original trajectory was prefixed by a short linear trajectory from the origin to the actual starting position at a running speed of 15 cm/s. The remaining minimal outer excursions were bounded by the environment's limits.

For each map cell, properties of grid cell firing like grid spacing, grid field width, gridness score, grid orientation, peak rate, mean rate, and inter-trial stability were computed for each trial; see **Post-processing** subsection in the Methods section. The mean and standard error of mean (SEM) of these properties within each independent population of map cells were obtained to observe various trends along the temporal rate gradient.

#### Case 1. Single cell: Rate gradient, fixed habituation, small-scale stripe cell input

To better understand model cell dynamics, we simulated the dynamics of a single category cell for different response rates (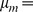
1, 0.5, 0.2, 0.1) at a fixed habituation rate 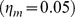 in response to a time-varying bottom-up input that is equivalent to the firing of a small-scale stripe cell in one of its stripe fields traversed at a speed of 10 cm/s in its preferred direction. Simulation results are provided in Figure 3.

#### Case 2. Spectral Spacing model: Rate gradient, fixed habituation, no noise, two stripe cell scales

**Figure 4:**
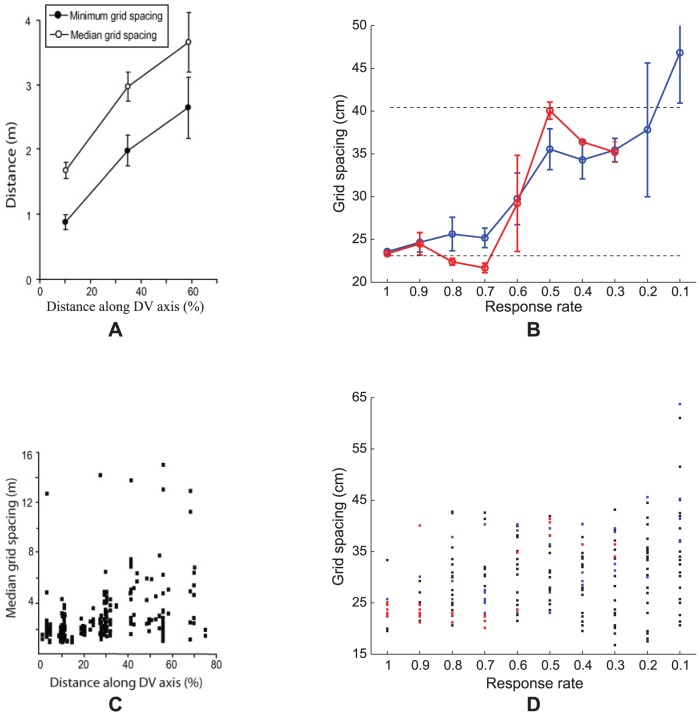
Grid spacing distributions. (A, C) Data [6] and (B, D) Case 2 simulation results regarding the distribution of grid spacing at different anatomical locations along the dorsoventral axis of MEC. Panels (A) and (B) provide error bar plots of grid spacing (mean +/− SEM). In (B), blue and red curves show grid spacing of learned map cells with gridness score >0 and those with gridness score >0.3, respectively, as a function of response rate 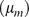 in the last trial. The two dashed lines parallel to the x-axis in (B) signify the two potential grid scales. In (D), grid spacings derived for all model map cells are shown for each response rate. Note that map cells with gridness score >0.3 are identified by red squares, and those among the remaining with gridness score >0 by blue squares, and the rest by black squares.

**Figure 5:**
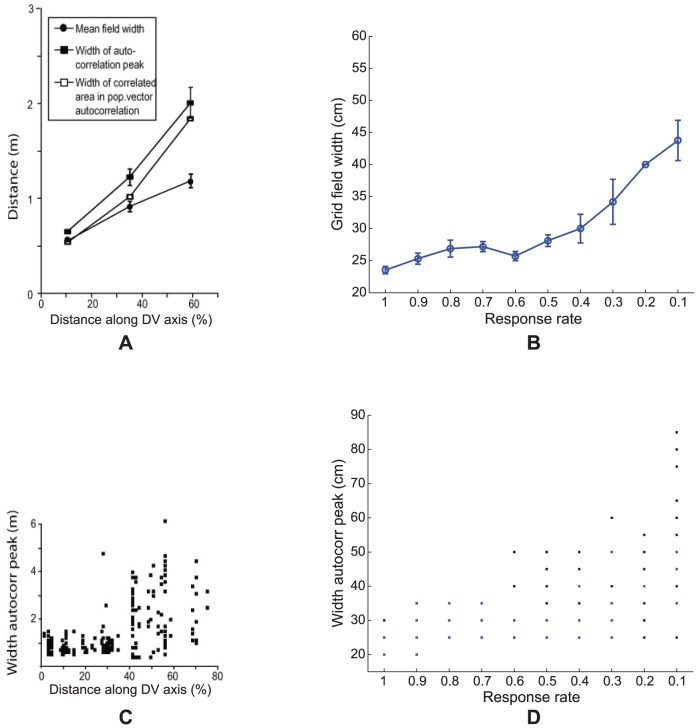
Grid field width distributions. (A, C) Data [6] and (B, D) Case 2 simulation results of the distribution of grid field width at different anatomical locations along the dorsoventral axis of MEC. Panels (A) and (B) provide error bar plots of grid field width (mean +/− SEM). In particular, panel (B) shows grid field width of learned map cells with gridness score >0 as a function of response rate 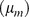 in the last trial. In (D), the width of the central peak in the spatial autocorrelogram of the rate map of all model map cells is shown for each response rate. Note that map cells with gridness score >0 are identified by blue squares, and the rest by black squares.

**Figure 6:**
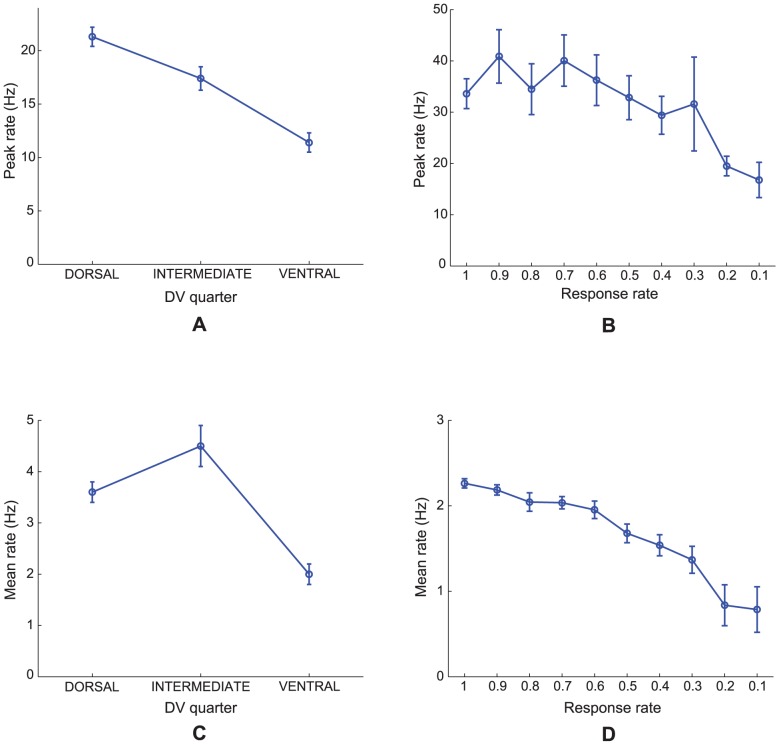
Grid cell peak and mean firing rates. (A, C) Data [6] and (B, D) Case 2 simulations regarding the (A, B) peak rates and (C, D) mean rates of grid cells (from their smoothed spatial rate maps) at different anatomical locations along the dorsoventral axis of MEC. Error bars in each panel show SEM. Model results are derived from learned map cells with gridness score >0 in the last trial.

**Figure 7:**
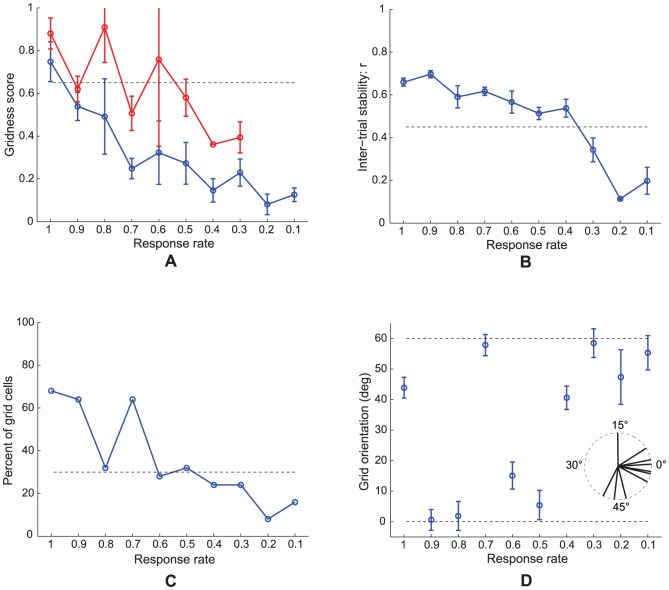
Case 2 results. Simulations for Case 2 of how (A) gridness score, (B) inter-trial stability, (C) percent of grid cells, and (D) grid orientation of learned map cells with gridness score >0 in the last trial vary as a function of response rate 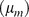. Panel (A) additionally plots gridness score of learned map cells with gridness score >0.3 in the last trial (red curve). Circular mean and standard deviation for grid orientation were calculated over the range [0°, 60°). Error bars in (A), (B), and (D) depict SEM. In (D), the inset provides a polar plot to depict mean grid orientation for various response rates, with the 360° range scaled for the 60° range of orientations. The dashed lines parallel to the x-axis in (A)–(C) signify corresponding experimentally measured values for adult dorsal grid cells [37], [38].

**Figure 8:**
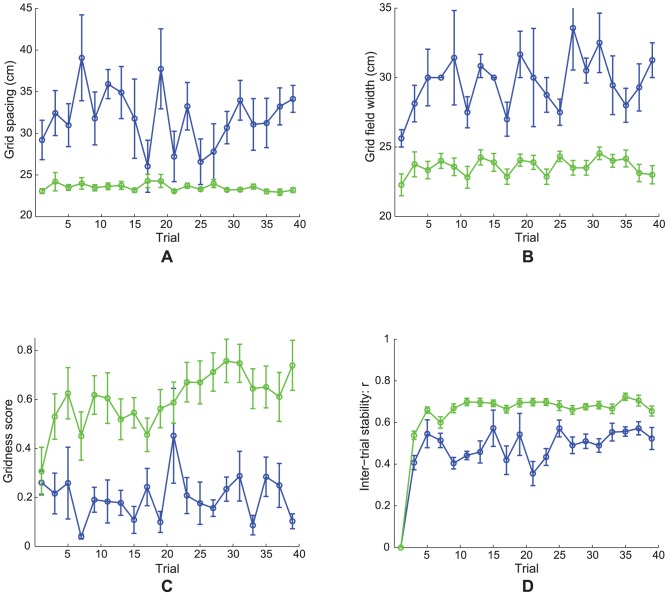
Model grid cell learning dynamics. Simulation results for Case 2 regarding how various measures of learned map cells with gridness score >0 vary as a function of number of learning trials for two representative response rates (dorsal: 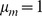 (green); ventral: 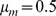 (blue)). Reported measures are (A) grid spacing, (B) grid field width, (C) gridness score, and (D) inter-trial stability. Error bars in each panel indicate SEM.

**Figure 9:**
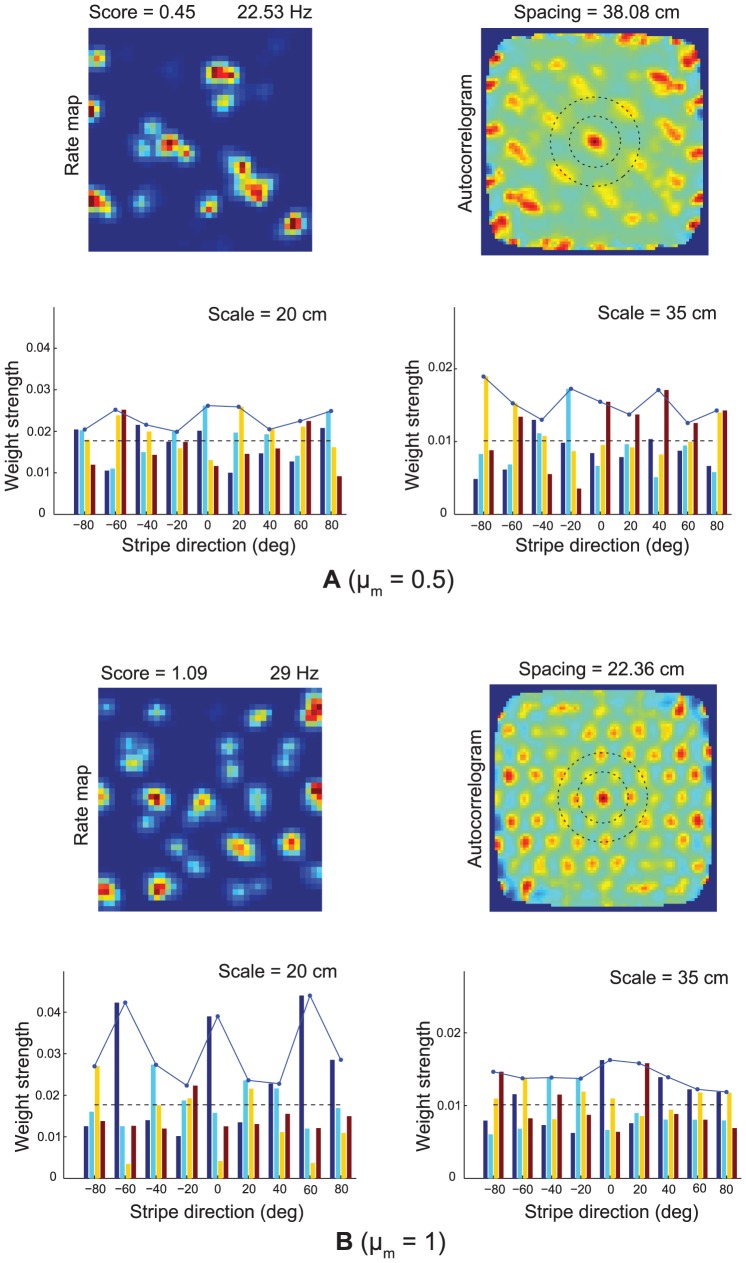
Pruned weights of inputs from multi-scale stripe cells. Simulations for Case 2 of the learned spatial fields and synaptic weights from stripe cells of two representative model grid cells, (A) one from a ventral location 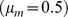, and (B) the other from a dorsal location 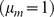, in the last trial. The top row in each panel shows the corresponding spatial rate map and autocorrelogram, with color coding from blue (min.) to red (max.). Note the gridness score and peak firing rate on the top of the rate map, and the grid spacing on top of the autocorrelogram. And the two dashed circles centered on the central peak in the autocorrelogram signify the two potential grid scales. The bottom row in each panel provides the adapted weights from the stripe cells of the two scales (20 cm, 35 cm) to the corresponding cell. Note the solid curves trace the maximal weight from each directional group of stripe cells, the dashed lines parallel to the x-axis signify the average weight level of the projections from the corresponding scale, and the y-axes for the two spatial scales have different weight scales.

**Figure 10:**
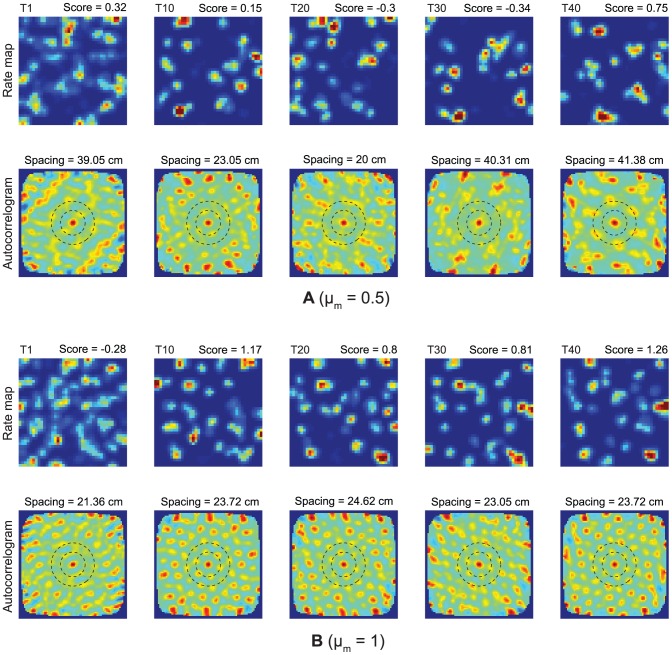
Spatial learning dynamics of two example model cells. Case 2 simulations showing the learned spatial fields of two representative model cells, (A) one from a ventral location 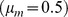, and (B) the other from a dorsal location 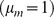, across the learning trials. The top and bottom row in each panel show the corresponding spatial rate map and autocorrelogram, respectively. Color coding from blue (min.) to red (max.) is used for these. Note the trial number (e.g., T1 = trial 1) and gridness score on top of each rate map, and grid spacing on top of each associated autocorrelogram.

The model was run with a gradient in response rate 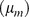 with the habituation rate fixed 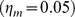 and in the absence of cellular noise 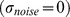. The gradient contained 10 non-interacting populations, corresponding to different anatomical locations along the dorsoventral axis of MEC. Each population contained 25 category cells. The only parameter that was varied across populations was the response rate 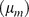, with values of 1, 0.9, 0.8, 0.7, 0.6, 0.5, 0.4, 0.3, 0.2, and 0.1. Two stripe spacings (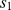 = 20 cm, 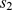 = 35 cm) were used. Stripe field width was assumed to vary in proportion to stripe spacing. In particular, the standard deviation of the stripe field Gaussian tuning was 8.84% of the stripe spacing (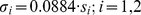; see Equation 1.4). The peak activity of large-scale stripe cells was chosen to keep the total activity of each stripe field for the different scales the same (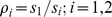; see Equation 1.4). In particular, as each stripe field is modeled by a Gaussian function, its total activity is given by 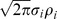 (see Equation 1.4), which is a constant (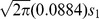) as 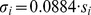 and 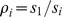. Simulation results are provided in Figures 4–10.

#### Case 3. Spectral Spacing model: Rate gradient, fixed habituation, no noise, two stripe cell scales, novel trajectories

**Figure 11:**
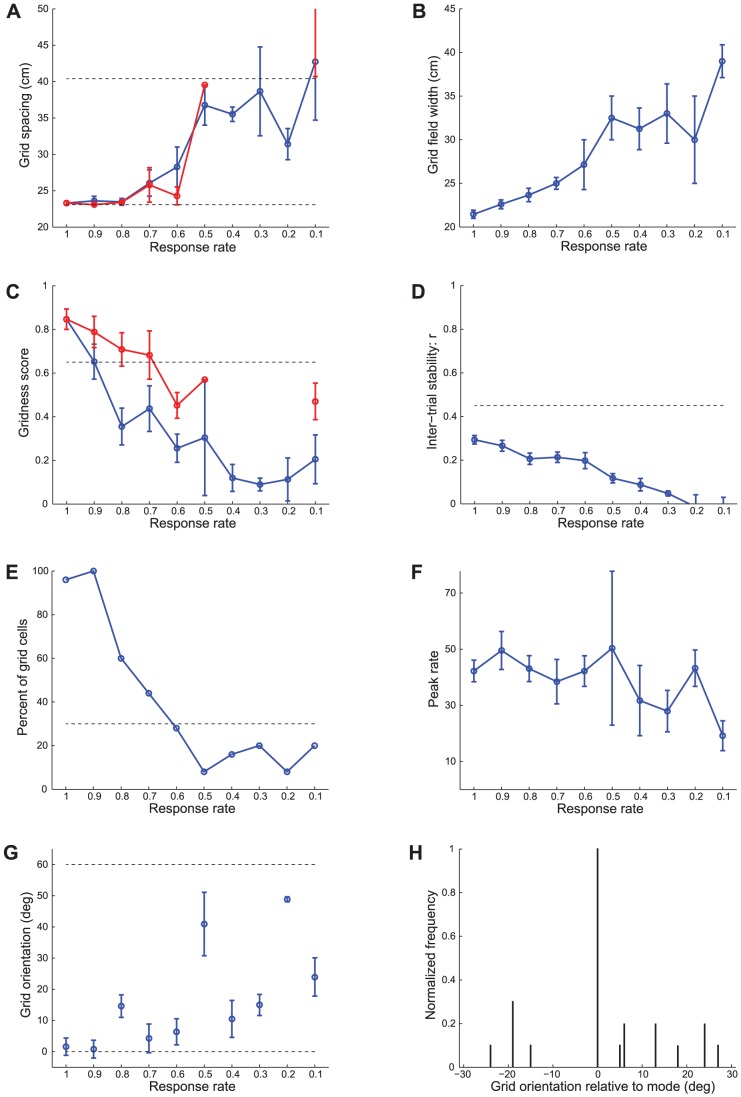
Case 3 results. Case 3 simulations in which the model animal runs along a novel realistic trajectory in each trial. The **Simulation Settings** subsection in the **Methods** section describes how various novel trajectories are generated from one realistic rat trajectory. Several measures of learned map cells with gridness score >0 in the last trial are shown as a function of response rate 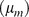: (A) grid spacing, (B) grid field width, (C) gridness score, (D) inter-trial stability, (E) percent of grid cells, (F) peak rate, and (G) grid orientation. Panel (H) shows the grid orientation distribution of map cells with gridness score >0 in the last trial for the dorsal most MEC population 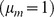. In (A) and (C), the red curves plot the corresponding measures of map cells with gridness score >0.3 in the last trial. The two dashed lines parallel to the x-axis in (A) signify the two potential grid scales. Dashed lines parallel to the x-axis in (C)–(E) signify experimentally measured values for adult dorsal grid cells [37], [38]. Error bars, present in all panels but (E) and (H) show SEM.

The same model equations (Equations 1.5–[Disp-formula pcbi.1002648.e107] 1.7) and input settings as in Case 2 were used, but the model animal traversed a novel realistic trajectory on each trial. See above for how these trajectories were chosen. Simulation results are provided in Figure 11.

#### Case 4. Spectral Spacing model: Rate gradient, fixed habituation, no noise, three stripe cell scales

**Figure 12:**
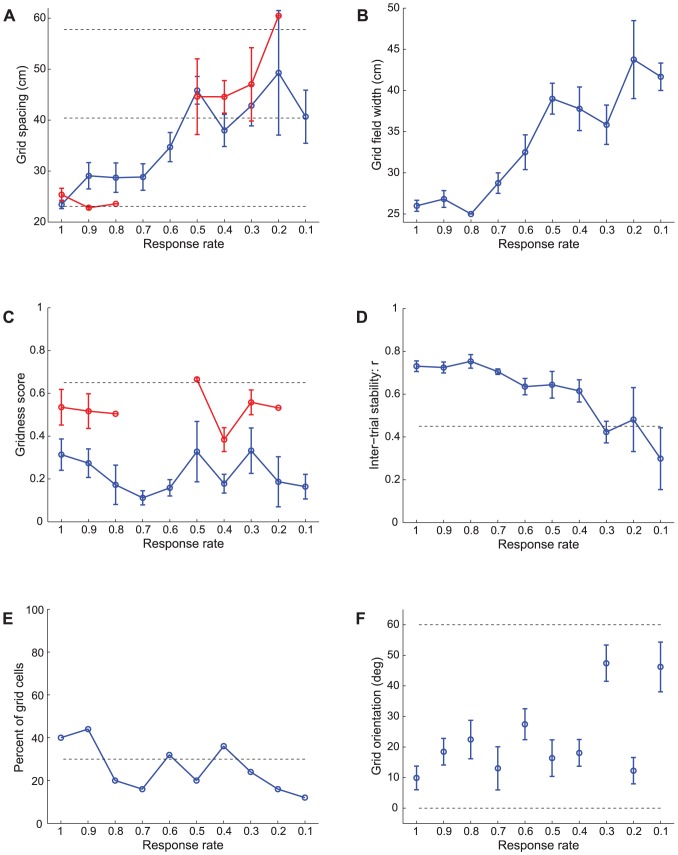
Case 4 results. Case 4 simulations in which the category cells receive projections from input stripe cells of three spacings (20 cm, 35 cm, and 50 cm). Several measures of learned map cells with gridness score >0 in the last trial are shown as a function of response rate 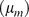: (A) grid spacing, (B) grid field width, (C) gridness score, (D) inter-trial stability, (E) percent of grid cells, and (F) grid orientation. In (A) and (C), the red curves plot the corresponding measures of map cells with gridness score >0.3 in the last trial. The three dashed lines parallel to the x-axis in (A) signify the three potential grid scales. Dashed lines parallel to the x-axis in (C)–(E) signify experimentally measured values for adult dorsal grid cells [37], [38]. Error bars, present in all panels but (E) show SEM.

This case also used Case 2 equations and settings. However, three stripe cell scales, with spacings of 20 cm, 35 cm, and 50 cm, were provided as inputs to map cells, for comparison with the two stripe scale simulations. All the other cases also simulated the model with two stripe cell scales. Simulation results are provided in Figure 12.

#### Case 5. Spectral Spacing model: Rate gradient, fixed habituation, noisy cells

**Figure 13:**
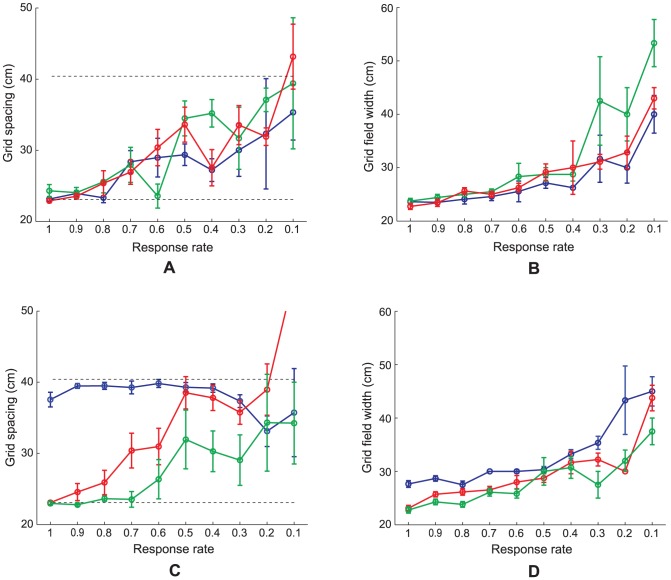
Results for Cases 5–10. (A, C) Grid spacing and (B, D) grid field width of learned map cells with gridness score >0 in the last trial as a function of response rate 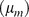 for different model and input variations: Case 5 (red curves in (A) and (B)); Case 6 (blue curves in (A) and (B)); Case 7 (green curves in (A) and (B)); Case 8 (blue curves in (C) and (D)); Case 9 (green curves in (C) and (D)); and Case 10 (red curves in (C) and (D)). See the **Simulation Settings** for detailed description of these various cases. The two dashed lines parallel to the x-axis in (A) and (C) signify the two potential grid scales. Error bars in each panel depict SEM.

In this case, the same equations and settings as in Case 2 (Equation 1.5–[Disp-formula pcbi.1002648.e107] 1.7) were used, but noise was injected into the membrane potential dynamics of all category cells 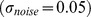 to test model robustness under noise. Illustrative results are shown in Figures 13A and 13B.

#### Case 6. Spectral Spacing model (variation 1): Rate gradient, fixed habituation, no noise

This is same as Case 2 except Equations 2.1–[Disp-formula pcbi.1002648.e137] 2.3 (variation 1) were employed for the model. Illustrative results are shown in Figures 13A and 13B.

#### Case 7. Spectral Spacing model (variation 2): Rate gradient, fixed habituation, no noise

This is same as Case 2 except Equations 3.1–[Disp-formula pcbi.1002648.e143] 3.3 (variation 2) were employed for the model. Illustrative results are shown in Figures 13A and 13B.

Cases 8–10 below additionally tested model robustness when stripe cell properties were varied. These results may help to search for stripe cells with particular properties in new experiments. In these simulations, peak activity and stripe field widths were varied for the two stripe cell spatial scales.

#### Case 8. Spectral Spacing model: Rate gradient, fixed habituation, no noise, constant stripe cell peak activity

This is the same as Case 2 except stripe cells of either scale had the same peak activity (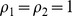; Equation 1.4). Figures 13C and 13D show illustrative results.

#### Case 9. Spectral Spacing model: Rate gradient, fixed habituation, no noise, constant stripe cell field width

This is the same as Case 2 except stripe cells had the same field width between the two scales (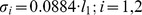; Equation 1.4). Figures 13C and 13D show illustrative results.

#### Case 10. Spectral Spacing model: Rate gradient, fixed habituation, no noise, constant stripe cell peak activity and field width

This is the same as Case 2 except stripe cells had the same field width and peak activity between the two scales (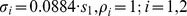; Equation 1.4). Figures 13C and 13D show illustrative results.

#### Case 11. Spectral Spacing model: Fixed rate, habituation gradient, no noise

**Figure 14:**
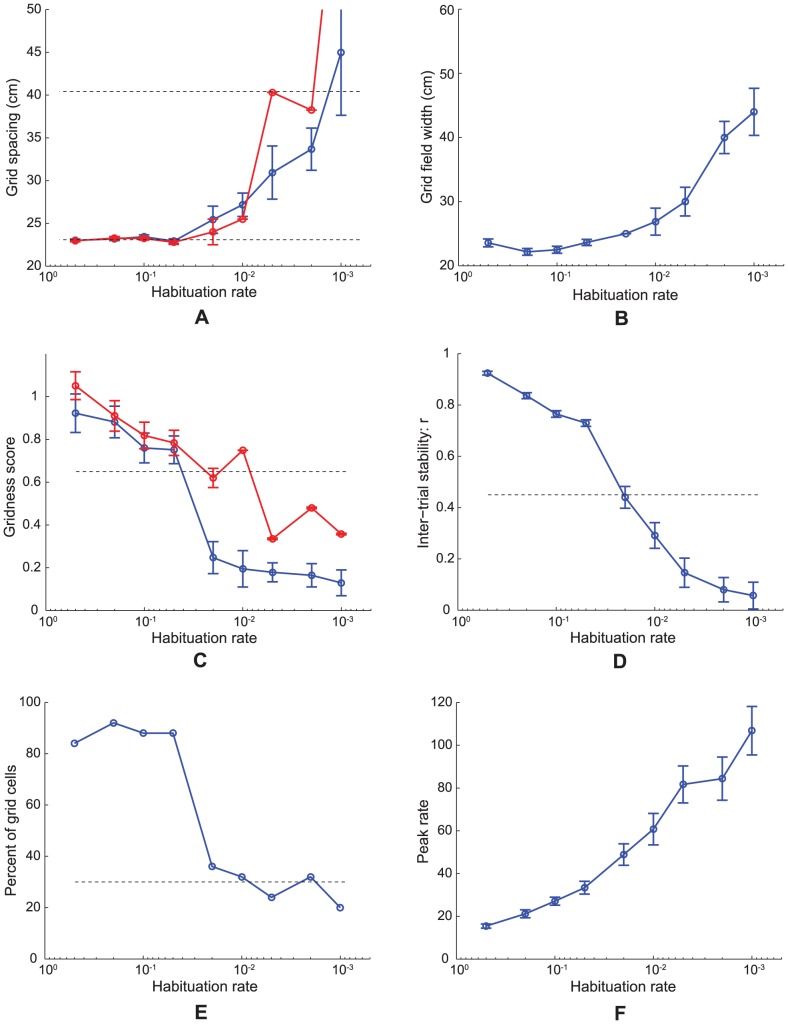
Case 11 results. Simulations for Case 11 in which it is the habituation rate that is varied with distance along the dorsoventral axis of MEC. Several measures of learned map cells with gridness score >0 in the last trial are shown as a function of habituation rate 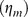: (A) grid spacing, (B) grid field width, (C) gridness score, (D) inter-trial stability, (E) percent of grid cells, and (F) peak rate. In (A) and (C), the red curves plot the corresponding measures for map cells with gridness score >0.3 in the last trial. The two dashed lines parallel to the x-axis in (A) signify the two potential grid scales. And the dashed lines parallel to the x-axis in (C)–(E) signify experimentally measured values for adult dorsal grid cells [37], [38]. Note the log10 scale of the x-axis in each panel. Error bars, present in all panels but (E), show SEM.

In this case, there was a gradient in habituation rate 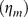 with the response rate fixed 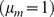 and no noise 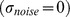. Here, nine non-interacting cell populations, each with 25 category/map cells, had different habituation rates 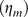, with the values of 0.5, 0.2, 0.1, 0.05, 0.02, 0.01, 0.005, 0.002, and 0.001. The settings for peak activities and stripe field widths were the same as those in the response rate gradient with no noise (Case 2). Simulation results are provided in Figure 14.

#### Cases 12 and 13. Membrane potential oscillations: Rate gradient, fixed habituation, noise; fixed rate, habituation gradient, noise

**Figure 15:**
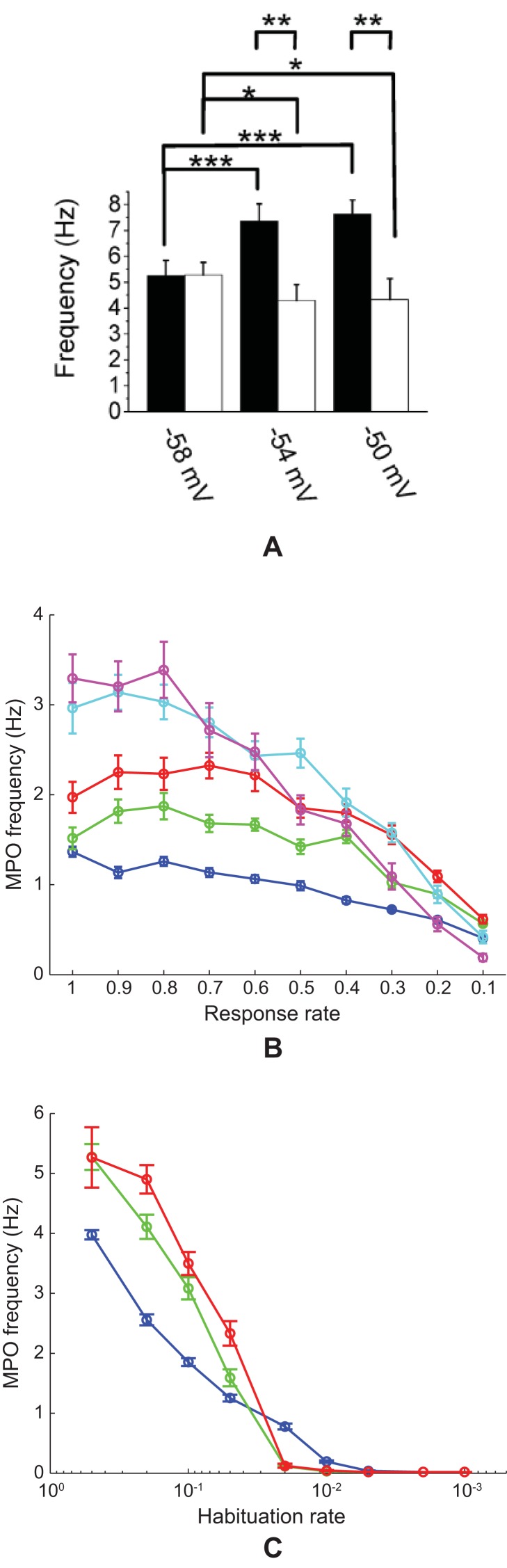
Membrane potential oscillations of medial entorhinal cells. (A) Data showing the frequency of subthreshold membrane potential oscillations (MPOs) in the dorsal (filled bars) and ventral (open bars) groups of rat MEC layer II stellate cells at three different mean membrane potentials [14]. See also Figure 1C in [13]. (B) Simulations of the frequency of MPOs of model category cells as a function of response rate 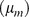, which is proposed to decrease along the dorsoventral axis of MEC, for current injections of different amplitudes (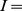
0.5 (blue); 1 (green); 1.5 (red); 2 (cyan); and 2.5 (magenta)). (C) Simulations of the frequency of MPOs of model category cells as a function of habituation rate 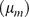 for current injections of different amplitudes (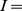
0.5 (blue); 1 (green); 1.5 (red)). Error bars in (A–C) indicate SEM.

**Figure 16:**
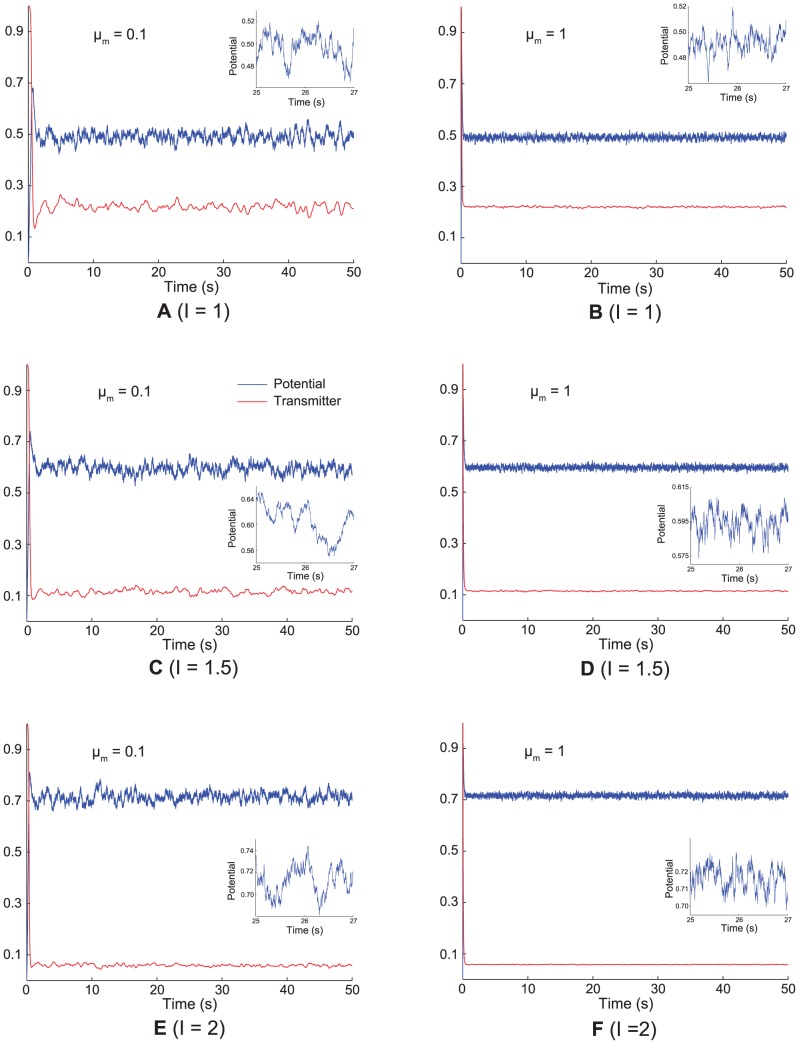
Model membrane potential oscillation traces. Simulations of membrane potential (blue curves) and habituative transmitter (red curves) traces in response to constant current injections of different amplitudes for a ventral category cell 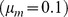 and for a dorsal category cell 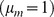, which are shown in the left and right columns, respectively. The three rows provide results for different current amplitudes: (A) and (B) for 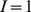; (C) and (D) for 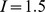; and (E) and (F) for 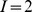. The inset in each panel zooms in on the noisy membrane potential fluctuation between 25 s and 27 s to highlight its relative frequency content.

MPOs with different periods were generated in response to a constant current input to simulate the *in vitro* studies of MEC layer II stellate cells at different locations on the dorsoventral axis (Figures 15A and 15A; [13], [14]). Two cases were simulated, one in which the response rate varied along the dorsoventral axis with the habituation rate fixed (Case 12; Figure 15B), similar to Case 2, and the other in which the habituation rate was varied with the response rate fixed (Case 13; Figure 15C), similar to Case 11 above. Constant current inputs of different amplitudes 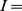
0.5, 1, 1.5, 2, and 2.5 in Equation 1.8 drove each category cell, in the absence of any intercellular interactions, for 50 s (Figure 16). Cellular noise 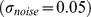 was added to help unmask damped oscillations [59].

## Results

### Effects of different response rates on individual cells

Figure 3 shows the results of the single cell simulation of Case 1 when that cell is given different response rates 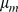 in Equation 1.5 in response to a stripe cell-like input (Figure 3A). Figure 3B shows the cell responses 
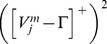
 when the on-center feedback term 
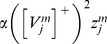
 is removed. As noted previously, self-excitatory feedback helps to contrast-enhance cell activity (compare Figures 3B and 3F). However, if the habituative gate 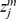 in Equation 1.5 is held constant at the value of one, then the outputs perseverate through time (Figure 3C). When transmitter gating is restored, the gates respond more slowly along the dorsoventral axis as their controlling cell activities do (Figure 3D), even if the habituation rate 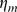 is the same across response rates, due to the activity-dependent term 
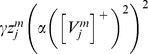
 in Equation 1.7. When the properties in Figures 3C and 3D are combined multiplicatively in the on-center feedback term 
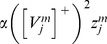
, it has a unimodal form that grows and decays more slowly as the cell response rate 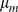 is decreased along the dorsoventral axis (Figure 3E). The cell output signals 
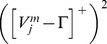
 along the axis inherit this variable-rate unimodal form (Figure 3F). In particular, cells exhibit a temporally delayed and broader response with a smaller peak activity for lower response rates. The higher the response rate, the faster is the activation of the membrane potential, allowing the cell activity to buildup to a higher level that is then gated off as quickly by the correlated change in the effective depletion rate of the transmitter. In this way, the habituative transmitter gating mechanism plays a role akin to a slow negative current that is activated by cell activity, much like the h-current 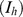 [60], and AHP currents [61].

The results of this simulation clarify how scale selection occurs (Cases 2–11). For a cell to respond with contrast-enhanced, or above-threshold, activity at any moment with the help of its self-excitatory feedback signal, its habituative transmitter needs to be at a sufficient high level. But each time the cell responds intensely, there is a collapse of the transmitter (Figure 3D), which takes longer to recover for slower response rates because of the increased duration of cell activity. This implies that, the slower the response rate, the longer the minimum temporal duration before the cell can again respond with above-threshold activity. In other words, ventral MEC cells, which have slower response rates in the model, favor periodic inputs that are presented with a longer temporal interval, and dorsal MEC cells, which have faster response rates, favor those that are presented with a shorter temporal interval.

This property directly explains learned scale selectivity for the case of a rat running forward at a constant speed on a linear track. Then dorsal MEC cells in the model respond better to inputs at periodic positions with relatively smaller spacings, while ventral MEC cells respond better to those with relatively larger spacings. However, the situation is more complicated when the rat navigates along the type of two-dimensional real trajectory used in our simulations, for which the running speed of the rat through time varies between 0 cm/s and 146.6 cm/s with a mean of 14.03 cm/s, a standard deviation of 9.8 cm/s, and a mean length of piecewise linear segments of only 0.9 cm. How different response rates selectively learn different spatial scales in response to such realistic trajectories is discussed in the next subsection.

### From different response rates to different learned spatial scales

Figure 4 compares neurophysiological data [6] with simulation results for Case 2 regarding the distribution of grid spacing at different anatomical locations along the dorsoventral axis of MEC. MEC grid cells exhibit periodic spatial firing fields whose spacing increases from the dorsal to the ventral ends (data: Figures 4A and 4C). Also, the spacing increases in variability along this axis. Brun and coworkers [6] remarked that the rat brain seems to allocate most of the grid cells to represent space at smaller scales, based on data that both intermediate and ventral MEC also have cells exhibiting periodic spatial responses with smaller spacings.

Emergent properties of model simulations (Figures 4B and 4D) emulate these data. Figure 4B plots grid spacing (mean +/− SEM) of learned map cells with gridness score >0 (see blue curve) and of those with gridness score >0.3 (see red curve) as a function of response rate, or equivalently the distance along dorsoventral axis, in the last trial. Figure 4D shows the distribution of spacing of all map cells as a function of response rate. Learned map cells with gridness score >0.3 are identified by red squares, and those among the remaining with gridness score >0 are identified by blue squares, and the rest by black ones. These results indicate that, despite non-stationary variations in running speed and in heading direction along a realistic trajectory in the open field, the response rates of the map cells select the spatial scale of input stripe cells to which the learned hexagonal grid firing fields maximally respond. Faster response rates can more effectively sample smaller stripe cell spatial periods, whereas slower response rates can do the same for larger stripe cell spatial periods, for reasons that are stated more precisely in the next paragraph. In this way, faster/dorsal MEC cells learned grid fields with smaller spacings, and slower/ventral MEC cells developed preference for larger grid spacings.

As noted earlier, for each input stripe scale considered separately, the most frequent and energetic activations of grid cells occur when sets of three stripe cells are coactivated whose preferred directions differ by 60° [20]. Now consider a dorsal map cell that becomes intensely active for the first time at some spatial position. During this first learning episode, the synaptic weights of its connections from stripe cells begin to get pruned to slowly match the normalized average input pattern. Given the faster dynamics of dorsal cells, this cell can again respond intensely to consistent stripe cell activations from either spatial scale at nearby positions as the animal moves around. Given the higher number of fields for a small-scale grid structure in a limited environment, and given the relatively lower peak activity of large-scale stripe cells, this dorsal cell has a higher likelihood of developing tuning to an appropriate set of stripe cells from the small scale. On the other hand, the slower dynamics of ventral cells prevents them, on average, from developing tuning to stripe cell coactivations from the small scale, because they tend to recur faster than the recovery rate of the ventral habituative transmitters. As a result, ventral cells that develop grid-like spatial selectivity gradually prefer stripe cell coactivations from the large scale. Increased variability in grid spacing for ventral cells may be understood as a manifestation of their weaker and temporally prolonged signal levels (Figure 3F), which cause broader regions of space to be incorporated into their developing selectivities. These results clarify how a gradient of temporal response rate leads to selective learning of the gradient of grid spatial scale, and are thus consistent with a recent study using HCN1 knockout mice regarding how manipulation of the anatomical gradient in intrinsic properties of stellate cells affects the gradient in grid scale [62].

Figure 5 shows neurophysiological data [6] and simulation results for Case 2 regarding the distribution of grid field width at different anatomical locations along the dorsoventral axis of MEC. MEC cells exhibit periodic spatial firing fields whose width increases from the dorsal to the ventral ends (data: Figures 5A and 5C). As for grid spacing, the grid field width also increases in variability along the axis. Model simulations (Figures 5B and 5D) match these data. An estimate for grid field width was obtained by computing the width of the central peak in the autocorrelogram where the correlation crosses zero. Figure 5B plots grid field width (mean +/− SEM) of learned grid cells as a function of response rate, or the distance along the dorsoventral axis, in the last trial. Figure 5D shows the distribution of field width of all map cells as a function of response rate. Learned grid cells are identified by red squares, while others by black ones.

### Decrease of peak firing rate along the gradient

Figure 6 shows neurophysiological data [6] and simulation results for Case 2 regarding the peak and mean firing rates of grid cells at different anatomical locations along the dorsoventral axis of MEC. Unlike grid spacing and grid field width, the *peak* firing rate of MEC cells decreases from the dorsal to the ventral ends (data: Figure 6A). There is also a negative trend for *mean* firing rate along the axis (data: Figure 6C). The model simulates and explains these data too by using the response rate gradient and normalized grid cell receptive fields, respectively. Figures 6B and 6D plot (mean +/− SEM) peak and mean firing rates, respectively, of learned grid cells as a function of response rate, or the distance along the dorsoventral axis, in the last trial. As we have already seen, faster response rates of map cells result in higher peak output activities (see Figure 3F). Given that the total area of the grid firing fields is roughly constant, or normalized, across spatial scales, a decrease in peak firing rate along the dorsoventral axis explains a decrease in mean firing rate.

### Multiple learned cell properties match neurophysiological grid cell data

Figure 7 shows how (A) gridness score, (B) inter-trial stability, (C) percent, and (D) grid orientation of learned grid cells in the last trial vary as a function of response rate for Case 2. Error bar plots (mean +/− SEM) are shown for gridness score, inter-trial stability, and grid orientation. Due to the regular hexagonal structure of grid cell spatial fields, grid orientation varies between 0° and 60°. Moreover, since grid orientations of 0° and 60° are identical, circular mean and standard deviation were calculated over the range of [0°, 60°). The hexagonal and periodic quality of the learned spatial firing fields, measured by the gridness score, decreases with response rate. Similarly, the spatial stability of the learned grid-like firing fields between consecutive trials, called the inter-trial stability, tends to decrease for slower response rates, with relatively poorer stability for the most ventral of the model MEC cells. The decrease in gridness score with distance along the model's dorsoventral axis coincides with the decrease in the proportion of learned grid cells. These three simulation results together suggest poorer and less stable pattern learning for ventral cells. Given the temporally delayed and broader output responses of ventral cells, the periods when the postsynaptic learning gate 
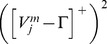
 in Equation 1.6 is positive do not correlate temporally as well with the activities of the triggering coactive stripe cells; compare the black curve in Figure 3A with the blue curve in Figure 3F. This situation results in a persistent recoding of the incoming weights for ventral cells as the trajectory is traversed, explaining their weaker inter-trial stability and gridness score measures. Fyhn and colleagues [3] have reported consistent data showing lower spatial stability for cells in ventromedial MEC compared to dorsolateral MEC (see their Figure 4J), but the recording enclosures used were relatively small to appropriately sample the large spatial scale of the ventral cells.

Model grid cells in each of the MEC local populations along the dorsoventral axis did not learn exactly the same grid orientation. However, given the recurrent inhibition among the category cells, the different hexagonal grid fields that are learned as a result of self-organization have minimal overlap among them, because of which all possible grid orientations are not equally likely. This can be understood as a consequence of how two sets of hexagonal grid fields of the same scale can have the least total overlap only when they share the same orientation. In SOM model simulations, clustering around a dominant orientation is often observed [20]. This occurs despite the lack of excitatory coupling among neighboring category cells, which helps to prevent a topographic map of grid spatial phases from being learned (data: [5]). Existing data on grid orientation at various dorsoventral locations are preliminary (Figure 2e in [5]; Supplementary Figure 4 in [63]), but seem to suggest a narrowly tuned distribution for grid cells recorded on the same tetrode. In our simulations, we observed that in general the spread of the orientation distribution is inversely correlated with the number of learned grid cells in the local population (see Figure 11H below for an example of a narrow learned orientation distribution). More systematic work aimed at ascertaining how the mean and spread of the grid orientation distribution vary along the dorsoventral axis is needed. The learned mean grid orientations along the response rate gradient, for Case 2, have a circular standard deviation of 9.87°, suggesting that grid orientations of different scales may not be similar. This is expected as the different local populations in our model do not mutually interact. The standard deviation of learned mean grid orientations for various response rates was 12.05° when a novel trajectory was used in each trial (see Figure 11G below), and was 12.76° when three input stripe cell spatial scales (20, 35, and 50 cm) were employed (see Figure 12F below).

Figure 8 presents simulation results for Case 2 regarding how various measures of learned grid cells vary as a function of number of learning trials, for two representative response rates (dorsal: 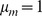; ventral: 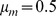). Reported measures are (A) grid spacing, (B) grid field width, (C) gridness score, and (D) inter-trial stability. Despite having to learn in response to two input stripe spatial scales, dorsal MEC cells (green curves in the four panels) pick out their spatial scale (grid spacing, grid field width) quickly and do not change their preference through time (Figure 8A). There is not much change in the inter-trial stability measure either (Figure 8D). Average hexagonal gridness quality of the learned grid firing fields for these model dorsal cells, however, shows gradual improvement over trials (Figure 8C). This is consistent with developmental data from rat pups regarding how emerging grid cells show significantly more change (improvement) in gridness score than in grid spacing [37]. Both the gradual improvement in gridness score of the grid cells with faster rates (Figure 8C, green curve) and the more rapid selection of grid spatial scales (separable curves in Figures 8A and 8B) reflect the tuning of bottom-up weights from stripe cells to grid cells. The rapid separation during learning of fast and slow rate grid cell properties can occur as soon as the different rates preferentially select stripe cells of compatible scale. The more gradual development of the gridness score for the faster response cells requires, in addition, detection and selection of the subset of projections from stripe cells of the smaller scale that are most frequently and energetically coactivated, and the suppression of less favorable correlations.

The ventral MEC cells (blue curves in the four panels) exhibit lower gridness scores (Figure 8C) and inter-trial stability (Figure 8D) measures that do change much through time, but show more fluctuation in their spatial measures through time (Figures 8A and 8B), although they exhibit higher values overall. As we have already discussed above, the slower dynamics of ventral cells explains their poorer learning and lower stability. The variability through time of their spatial scale may also be related to their energetically smaller and temporally broader signal levels (Figure 3F).

### Learned adaptive weights are scale-selective

Figure 9 shows Case 2 simulations of learned spatial fields and synaptic weights from stripe cells of two representative model grid cells, (A) one from a ventral location 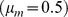, and (B) the other from a dorsal location 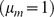, in the last trial. The spatial autocorrelograms of the rate maps (see top right in each panel of Figure 9) make clear the underlying spatial scale of the grid fields. Consistent with the exhibited spatial scales, only the maximal adapted weights from each stripe cell ring attractor for the corresponding scale show local peaks whose preferred directions differ by 60°. These results are consistent with the explanation given for the scale differences in Figure 4. Once again, the temporal response rate constrains the spatial scale of the stripe cells that can succeed in shaping and driving the emerging grid cells.

Figure 10 shows simulation results for Case 2 regarding the learned spatial fields of two representative model cells, (A) one from a ventral location 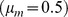, and (B) the other from a dorsal location 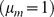, across learning trials. These illustrate in greater detail the relatively poorer inter-trial stability and higher preference for larger spatial scales for ventral MEC cells.

### Novel realistic trajectory on each trial

Figure 11 presents simulation results for Case 3 in which the model animal runs along a novel realistic trajectory in each trial. Several measures of learned map cells in the last trial are shown as a function of response rate 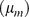; namely, (A) grid spacing, (B) grid field width, (C) gridness score, (D) inter-trial stability, (E) percent of grid cells, (F) peak rate, and (G) grid orientation. Additionally, panel (H) shows the grid orientation distribution of learned map cells for the dorsal most MEC population 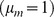. These results demonstrate that the ability of the Spectral Spacing model to solve the stripe scale selection problem (Figures 4–7) is not tied to the particular navigation trajectory (see Figure 1B) that was used for Case 2. The main quantitative differences with Case 2 are a relatively higher gridness score and proportion of learned grid cells, but lower inter-trial stability. These can be interpreted as consequences of, respectively, experiencing more hexagonal grid exemplars, and undergoing more persistent recoding of synaptic weights from stripe cells as a result of denser environmental coverage [20].

### Three input stripe cell scales

Figure 12 presents simulation results for Case 4 in which the category cells receive projections from input stripe cells of three spacings (20 cm, 35 cm, and 50 cm). These stripe spacings were chosen such that their ratio (1∶1.7∶2.5) matches that of the smallest three grid spacings across rats [63]. Several measures of learned map cells in the last trial are shown as a function of response rate 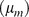: (A) grid spacing, (B) grid field width, (C) gridness score, (D) inter-trial stability, (E) percent of grid cells, and (F) grid orientation. These results demonstrate that the Spectral Spacing model can also select from among three scales of input stripe cells for grid scale gradient learning. Development of even larger grid scales will require realistic trajectories of rats in much bigger environments (i.e., much greater than 100 cm×100 cm). The main quantitative differences with Case 2 are a relatively lower gridness score and proportion of learned grid cells, but higher inter-trial stability. More input stripe cells, due to the additional scale, reduce the effective rate of change in the bottom-up weights to map cells (see Equation 1.6). This reduced plasticity correlates with more stability, but slows down the improvement in hexagonal gridness of the spatial fields of the developing map cells.

### Robustness of learning in model and input variations

Figure 13 summarizes for other model and input variations the learned grid spacing (Figures 13A and 13C) and grid field width (Figures 13B and 13D) of grid cells in the last learning trial as a function of response rate. These model and input variations include injection of noise into membrane potential dynamics of map cells (Case 5); changes to the learning law and how the habituative gating mechanism operates (Case 6; Equations 2.1–[Disp-formula pcbi.1002648.e137] 2.3); a different signal function governing output activities of map cells (Case 7; Equations 3.1–[Disp-formula pcbi.1002648.e143] 3.3); stripe cells with the same peak activity between the two scales (Case 8); stripe cells with the same field width between the two scales (Case 9); and stripe cells with both the same peak activity and field width between the two scales (Case 10). In all model variations but Case 8, which we discuss below, learned grid spacing and field width vary as in the data along the dorsoventral axis of MEC. Simulations for Cases 5, 6, and 7 are shown in Figures 13A and 13B by blue, green, and red curves, respectively, and simulations for Cases 8, 9, and 10 are shown in Figures 13C and 13D by blue, green, and red curves, respectively.

### Key role of normalization in generating the spatial gradient

In Case 8, unlike the data, dorsal cells learned hexagonal grid fields derived from large-scale stripe cells. This case imposes the same peak activity across both small-scale and large-scale stripe cells. Thus, the stripe cell receptive fields are not normalized across scales, and the large-scale stripe cells have a competitive advantage since they are sampled by map cells for a longer time. This advantage seems to be sufficient for them to win over small-scale stripe cells with the same peak activity, despite the lower frequency of their favored coactivations across space (7 for the stripe spacing of 35 cm, compared to 23 for the stripe spacing of 20 cm, in a 100 cm×100 cm field), in driving the learning of large-scale grid cells even for faster response rates. Thus, if stripe field widths increase with stripe spacing, similar to grid cells [6], this result suggests a need for a concomitant decrease in peak activity for stripe cells; in other words, stripe cell receptive fields need to be normalized.

### Normalized receptive fields as a general design principle

Normalized receptive fields occur in many other examples of multi-scale processing in the brain, and may be a general principle of brain design. The general design theme is how to achieve selective processing across multiple scales, so that the largest scales do not always win the competition to represent incoming data. Normalization ensures that the degree of brain commitment covaries with the amount of evidence for that choice [64]. In particular, normalized multiple scales help to ensure: speed-selective processing of visual motion, with larger scales responding selectively to faster speeds [65]; depth-selective perceptual grouping wherein larger oriented filters can represent nearer depths but smaller filters only represent farther depths [66]; and length-selective processing of speech wherein longer sequences of items stored in working memory can selectively activate list chunks that represent these longer sequences, which in turn suppress the activity of list chunks that respond to shorter sequences [64], [67], [68].

### Response rate gradient, but not habituation gradient, explains all the data

Figure 14 shows the simulation results for Case 11 in which it is only the habituation rate 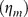 that is varied. Several measures of the learned grid cell firing are shown, namely, (A) grid spacing, (B) grid field width, (C) gridness score, (D) inter-trial stability, (E) percent of grid cells, and (F) peak firing rate, as a function of habituation rate. All measures except the peak firing rate are consistent with those obtained from a response rate gradient (Figures 4, 5, and 7). The peak firing rate increases as the habituation rate decreases with distance from the dorsal end (Figure 14F), in contrast with Figure 6B, where the peak firing rate decreases with response rate. The increase in peak output activity for map cells with habituation rate decrement can be understood as follows: With the response rate fixed, slower habituation rates result in slower collapses of transmitter, which are thus increasingly unable to counter the amplifying effect on grid cell activity of the self-excitatory feedback signal. These observations allow us to single out, in our model, the response rate as the parameter that most likely enables the learning of the dorsoventral gradient in grid cell spatial scale. Experimental studies [62], [69] have reached a similar conclusion that relatively slower temporal summation by ventral MEC cells most likely accounts for their increased spatial scale.

### MPO frequency gradient emerges from response rate gradient

As noted in the **Introduction** section, *in vitro* studies have showed that layer II MEC stellate cells exhibit subthreshold MPOs, in response to steady current injection, whose temporal period increases from the dorsal to the ventral end of MEC (Figure 15A), thereby correlating with the observed gradient in spacing and field width of grid cell spatial responses [13], [14]. In our model, when a steady current is injected into each category cell in the absence of any intercellular interactions (Equation 1.8), an MPO is generated with a frequency that tends to covary with both the response rate (Figure 15B) and the habituation rate (Figure 15C) for various current amplitudes 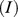. Our results suggest that, although there is a correlation between the gradient of MPO frequency and the gradient of grid cell spacing and field width, there is no direct causal link between them. The MPO frequency gradient is just an emergent property that results from model dynamics that control grid cell learning and activation. In particular, when a model category cell depolarizes in response to current injection, the positive feedback signal amplifies cell activity. This amplification increases the activity-dependent rate of inactivation of the habituative gate (Equation 1.7), which thereby gates off the amplification, causing the cell to become less active. Since the habituative gate is activity-dependent, it then recovers, and the cycle repeats leading to oscillations in the membrane potential. A faster response rate leads to faster amplification, habituation, and recovery; thus, to a faster oscillation (Figure 15B). A faster habituation rate, even for fixed response rate, has a similar effect (Figure 15C) because the habituative gate again collapses more quickly, thereby gating off the amplification more quickly, which in turn enables the transmitter to recover more quickly. Figures 16A, 16C, and 16E summarize simulations of membrane potential and habituative transmitter traces in response to current injections of different amplitudes for a ventral MEC cell with a slow response rate 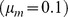, and Figures 16B, 16D, and 16F summarize simulations for a dorsal MEC cell with a fast response rate 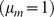. Note the faster MPO for the faster response rate.

Yoshida and coworkers [14] studied the effect of depolarization on the frequency of subthreshold MPOs within single MEC layer II stellate cells at different locations on the dorsoventral axis (Figure 15A). They reported that the MPO frequency of dorsal cells tends to increase with depolarization, and that of ventral cells tends to decrease. However, these positive and negative effects at ventral and dorsal locations are statistically significant only if the low-power broadband MPOs at the most hyperpolarized levels are included in the analysis. These data are consistent with our simulations of the effect of current amplitude on MPO frequency, presented in Figures 15B and 15C. In the Spectral Spacing model, increased current amplitude tends to cause a faster recovery of the cell potential in each MPO cycle after the phases of amplification and habituation. However, larger current amplitudes, with their resultant higher mean membrane potentials and lower mean habituative transmitters, cause relatively lower amplitude oscillations about the mean levels (Figure 16). This happens because the habituatively gated self-excitatory feedback term 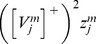, which controls the oscillatory dynamics, decreases with increasing current amplitude 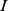; see Equation 1.8. Cellular noise begins to obscure the general positive effect of current amplitude on the frequencies of such oscillations, especially for slower response rates and habituation rates. This explains the saturation effect of depolarization on MPO frequency at all locations along the dorsoventral extent of MEC, and the apparent negative trend of MPO frequency with depolarization for ventral cells.

The simulation results in Figures 4–16 together clarify how all the observed gradient properties of grid cells can be explained as emergent properties of a gradient of response rates in a suitably defined SOM.

## Discussion

### Stripe cells

Our model, and [23] before us, propose that stripe cells and head direction cells use 1-D ring attractor networks to perform path integration in response to linear and angular velocity inputs, respectively. This proposal suggests that the brain parsimoniously uses a similar design to integrate both types of velocity inputs. Different stripe scales may, for example, result from different gains of linear velocity in controlling the speed of revolution of the activity bump along the ring of cells.

It remains an open experimental question as to how many spatial scales of stripe cells may exist. The current simulations show how the dorsoventral gradient of grid cell spatial scales may self-organize in response to either two or three stripe cell scales. In principle, it is possible that there are as many scales of stripe cells as there are scales of grid cells. In particular, are the stripe cells, in parasubiculum [24] or another parahippocampal subregion, arranged with respect to spatial scale in a manner similar to the grid scale gradient in layer II of MEC? It is also an open question as to whether the seemingly constant ratio (1∶1.7∶2.5) of the three smallest grid spacings across rats [63] is mirrored in the stripe cell layer, or emerges through learning from the response rate gradient across grid cells. Even if there are as many stripe cell scales *in vivo* as grid cell scales, the problem of how entorhinal cells learn to select their spacing from various scales of input stripe cells needs to be addressed, since they would likely receive inputs from a significant portion of the stripe cell gradient, comprising at least a few scales if not all, similar to how principal cells at an arbitrary dorsoventral location in the hippocampal formation receive projections from about a quarter of the dorsoventral extent of superficial MEC [70].

### Why are grid cells needed?

Our model proposes how path integration information is hierarchically processed in the medial entorhinal-hippocampal system (stripe cells to grid cells to place cells) to convert a stripe cell population code that implicitly represents an animal's position using multiple small spatial scales into a place cell code in which a single place cell can explicitly represent spatial position in large environments. The intermediate stage of grid cells converts input stripe cell signals into a form conducive to the learning of such unimodal place cell spatial fields, which thereby significantly increase the scale of spatial representation compared to the inducing grid cells. Simulations in [11] illustrate the possibility that the hippocampal spatial scale may be as large as the least common multiple of the inducing grid cell scales. The Spectral Spacing model shows, in turn, how the gradient of inducing grid cell spatial scales can be learned as a result of a response rate-based selection process.

Can place cells be learned directly from stripe cells, without the intervention of hexagonal grid fields? The presence of the animal at a given spatial position strongly activates just one or few stripe cells in each directional ring attractor. So, a unimodal spatial field at that position could be learned, in principle, if a map cell could become tuned to the combination of all these coactive stripe cells across directions and scales. However, such input combinations are not favored by the self-organization process because they occur only at single positions in the environment, as opposed to the multiple positions at which the stripe cell combinations suitable for hexagonal grid fields are activated. As we mentioned above, map cell learning at both the grid cell and place cell levels is naturally sensitive to both the energy and frequency of input coactivations. How, then, are place cells learned, given they are activated only at single positions in the environment? If inputs to a SOM come from comprise grid cells of multiple spatial scales, then sets of co-active grid cells involving a greater number of scales are more likely to gain control of hippocampal map cells [20]. However, grid cell coactivations from two or more scales do not occur more than once in typical-sized environments [11], especially because grid scales differ by non-integer ratios [63].

### Response to wide variations in average running speed

The spacings of grid fields in our model are adaptively selected based on cell response rate, which is inversely correlated with the minimum temporal duration between two episodes of intense activity. Therefore, it is important to discuss how the learned grid cells may respond if an adult animal were to run around an environment with a mean speed that is higher or lower than when the grid cells are learned during development. However, these extreme speed cases may be relevant only for theoretical purposes because of two reasons; namely, the distribution of running speeds in the realistic trajectory, used for our simulations, is relatively broadly tuned with a standard deviation of 9.8 cm/s, and existing relevant data indicates that the average running speed of rats increases by just ∼2 cm/s from P16 to adulthood (see Supplementary Figure 1F in [37]). In both data and model (Figures 4C and 4D), neighboring grid cells exhibit a spectrum of spacings in their spatial responses, especially with more distance along the dorsoventral axis, and our simulations show that only a subset of them can be classified as grid cells (Figure 4D). It may thereby be that high mean speeds favor learned map cells with larger spacings at a given dorsoventral location in order to express an appreciable hexagonal grid spatial activity pattern, whereas low mean speeds may favor those with smaller spacings. This possibility in the model is related to how the excitability of a map cell is dependent on the level of the habituative transmitter, whose depletion and recovery dynamics are in turn controlled by the response rate variable. The firing rate [4] and inter-burst frequency [71] of grid cells are known to vary in proportion to running speed. These data suggest that the response rates of MEC layer II cells *in vivo* may be modulated by running speed, because of which the slope and intercept of the dorsal-ventral gradient in grid spacing may not be significantly altered in response to very fast or slow running speeds. It would be instructive to explicitly test this prediction.

### Learning a stable gradient of grid cell scales: Top-down attentional matching

Our model simulations illustrate how gradients in intrinsic properties such as membrane potential oscillation frequencies of stellate cells along the dorsoventral axis of MEC layer II may arise from the same response rate mechanism that constrains the learning of the gradient of grid cell spatial scales. This prediction is consistent with data of [72], which showed that the anatomical gradient in intrinsic properties of MEC layer II stellate cells exists before the rats begin to explore their spatial environments for the first time.

Boehlen and colleagues [72] also reported, using sharp microelectrode recordings, that the peak frequency of subthreshold MPOs in the MEC increases as juvenile rats age into adults (see their Figure 3B), though such an age-dependent change was not seen in patch clamp recordings (see their Figure 3D). In contrast, studies that investigated the development of grid fields from postnatal (P16) to adult stage [37], [38] did not report any age-dependent variation in spatial periods of grid cells. This lack of change in spatial scale could be due to mechanisms that dynamically stabilize grid fields after they form. In particular, the spatial stability of grid cell receptive fields may require top-down feedback from place cells [73]. Such top-down interactions, among other memory-stabilizing processes, may dynamically buffer previously learned connections in the entorhinal-hippocampal hierarchy against the effects of a response rate change.

Indeed, place cell selectivity can develop within seconds to minutes, and can remain stable for months [74]–[77]. Such a combination of fast learning and stable memory is often called the *stability-plasticity dilemma* [44], [78]. Grossberg [40] showed that SOMs, by themselves, cannot solve the stability-plasticity dilemma in environments whose input patterns are dense and non-stationary through time, as occurs regularly during real-world navigation. In response to such inputs, learned categories can be persistently recoded by new inputs. However, SOMs augmented by learned top-down expectations that focus attention upon expected combinations of features can do so.

Adaptive Resonance Theory, or ART, was introduced in [79] to show how to dynamically stabilize the learned memories of SOMs. In ART, learned top-down expectations match bottom-up input patterns to focus attention upon expected combinations of critical features, drive fast learning of new, or refined, recognition categories that incorporate these critical feature patterns into their learned prototypes, and dynamically stabilize established memories. Grossberg [80] proposed how such attentive matching mechanisms from hippocampal cortex to MEC may stabilize both learned grid and place cell receptive fields. Besides helping to account for why the spatial scales of grid cells are maintained despite changes in intrinsic cellular properties as development proceeds [72], the incorporation of top-down connections from place cells to grid cells may also help to improve the spatial stability of learned grid fields (Figures 7B and 11D).

Experimental data about the entorhinal-hippocampal system illustrate how the predicted properties of top-down expectations and attentional matching may play a role in spatial learning and memory stability. Kentros and colleagues [81] reported that “conditions that maximize place field stability greatly increase orientation to novel cues. This suggests that storage and retrieval of place cells is modulated by a top-down cognitive process resembling attention and that place cells are neural correlates of spatial memory”, and that NMDA receptors mediate long-lasting hippocampal place field memory in novel environments [82]. Morris and Frey [83] proposed that hippocampal plasticity reflects an “automatic recording of attended experience.” Bonnevie and colleagues [73] showed that hippocampal inactivation causes grid cells to lose their spatial firing patterns.

In summary, our model here and in [20] of grid and place cell learning uses self-organizing maps (SOMs). *Every* SOM can exhibit catastrophic forgetting in response to a dense non-stationary input environment. ART top-down matching and attentional focusing mechanisms can dynamically stabilize learning in any SOM; that is, they solve the *stability-plasticity dilemma*. It is known that grid and place cells solve the stability-plasticity dilemma. Thus, our SOM model is incomplete, but *because* the model uses SOMs, there is a clear path for completing it, unlike other kinds of grid cell models, such as oscillatory interference and 2-D attractor models, which have not yet shown how the learning of their grid cells happens, and further how this learning may be dynamically stabilized (see subsection below on **Other grid cell models**). The nature of our model's incompleteness clarifies data about how and when deformations in grid cell receptive fields do occur [73]. Finally, there are important data from several labs (e.g., Berke, Kandel, and Morris) showing the kinds of attentional, learning, and oscillatory dynamics that ART predicts for the stabilization of place cell learning. Our model hereby clarifies an important conceptual link between these data about place cells and data about attention, learning, memory, and oscillations in grid cells.

### Membrane potential oscillations in MEC layer II stellate cells to steady input current

More work needs to be done to study how the response rate gradient and the habituative gating mechanism in our model relate to the HCN and leak potassium channels, which control the varied temporal integrative properties of MEC layer II stellate cells [19], [84], [85]. However, the manner in which MPOs arise in our model category cells is similar to how subthreshold MPOs in these stellate cells are known to occur based on the concerted action of a positive and a negative current [86]; in particular, persistent sodium (NaP) current and hyperpolarization-activated cation current 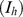, respectively [60]. The habituative gating mechanism is similar to how AHP currents control adaptation and refraction in proportion to recent cell activity. Indeed, the proposed gradient in cell response rates, which modulates habituative gate dynamics, is consistent with data showing an increase in the recovery time constant of mAHP currents along the dorsoventral axis of MEC [87].

### Predictions

The model suggests several predictions regarding the development of grid cells at different anatomical locations along the dorsoventral axis of MEC as young animals begin to navigate for the first time. These predictions are tempered by the awareness that the model does not yet incorporate various known mechanisms, such as top-down matching and attentional mechanisms from hippocampus, that may influence model properties, notably their malleability after the predicted dynamical stabilization of grid field structures sets in due to attentive matching.

Existing empirical studies on the development of grid cells [37], [38] have not looked for differences in the learning dynamics of grid cells across spatial scales. Model simulations suggest that lower proportions of grid cells, lower gridness scores, lower spatial stability, and higher variability in grid spacing through time may be found at more ventral locations of MEC.

### Neural relativity by Spectral Spacing and timing: Homologous space and time representation in entorhinal-hippocampal circuits and episodic memory

The Spectral Spacing model illustrates how control by a single rate parameter can determine a gradient of grid cell spatial scales in response to inputs from multiple stripe cell spatial scales. Multiple small grid cell scales can then be adaptively combined in the hippocampus to generate place cell scales that are large enough to support spatial navigation [11], [20]. A similar strategy for temporal coding seems also to occur in the brain: Previous modeling [50]–[52] has shown how control by a single rate parameter can determine a gradient of small temporal scales that can be adaptively combined in the hippocampus to generate temporal scales that are large enough to bridge temporal gaps between stimulus and response, such as those that occur during trace conditioning and delayed non-match to sample experiments. As we noted earlier, this latter type of model is called a Spectral Timing model.

In support of this prediction, MacDonald and coworkers [88] have reported hippocampal “time cells” that have all the properties required to achieve spectral timing; in particular, “… the mean peak firing rate for each time cell occurred at sequential moments, and the overlap among firing periods from even these small ensembles of time cells bridges the entire delay. Notably, the spread of the firing period for each neuron increased with the peak firing time …” The correlation of the peak firing time with the spreading of the firing period is called a Weber law, and is one of the dynamical signatures of spectral timing. It remains to be shown whether the spectrum of time cells arises from a gradient in a single rate parameter. A biophysical interpretation of this rate parameter in terms of calcium dynamics in the metabotropic glutamate receptor system has been given for the case of spectral timing in the cerebellum [89]. The most parsimonious prediction is that a similar mechanism holds in all cases of spectral timing throughout the brain. To the present, spectral timing has been modeled in the hippocampus, cerebellum, and basal ganglia [90].

Thus, dorsoventral gradients in single rate parameters within the entorhinal-hippocampal system may create multiple small spatial and temporal scales that can be fused into larger spatial and temporal scales in the hippocampal cortex that are large enough to control adaptive behaviors. The mechanistic homology between these spatial and temporal mechanisms suggests why they may occur side-by-side in the medial and lateral streams through entorhinal cortex into the hippocampus. In particular, spatial representations in the *Where* cortical stream go through postrhinal cortex and medial entorhinal cortex on their way to hippocampal cortex, and object representations in the *What* cortical stream go through perirhinal cortex and lateral entorhinal cortex on their way to hippocampal cortex [2], [91]–[94], where they are merged. This unity of mechanistically homologous space and time representations may be summarized by the term “neural relativity”. The existence of such computationally homologous spatial and temporal representations in the hippocampus may help to clarify its role in mediating episodic learning and memory. Indeed, investigators since Tulving [94]–[98] have suggested that each episode in memory consists of a specific spatio-temporal combination of stimuli and behavior, and discussed evidence supporting this claim.

### Differences with GRIDSmap model

This subsection highlights and justifies differences between the GRIDSmap model [23] and the current Spectral Spacing model.

First, we introduced a threshold in the signal function that transforms membrane potentials of map cells into their output activities, which both govern the recurrent inhibitory interactions and gate the competitive adaptation of corresponding bottom-up weights (see parameter 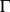 in Equations 1.5 and 1.6). This helps to ensure the following properties [20]:

- Non-zero inhibitory signals do not arise from noisy or non-optimally activated map cells. Computationally, this prevents low levels of activity-dependent plasticity due to cumulative inhibition from several noise-activated map cells. A threshold of 0.1 is sufficient to prevent this problem even as the number of map cells is scaled up over a wide range (5–200).
- Increased stability of learned spatial fields as the bottom-up weights do not adapt in response to noisy or non-optimally activated map cells.

Second, we initialized the pre-development synaptic weights of the connections from stripe cells to grid cells (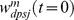 in Equation 1.6) using a uniform distribution between 0 and 0.1. The mean of these initial weights (0.05) is higher than that (0.0075) used in [23]. This helps to ensure that each entorhinal map cell in a larger population (>>5 in [23]) is activated at least somewhere in the environment, and thereby participates in activity-dependent learning to likely emerge as a grid cell [20]. Map cells with initial weights from stripe cells that are low, or with those that do not closely match any input pattern during spatial navigation, cannot adapt enough to contribute towards spatial representation.

Third, the inactivation and recovery dynamics of the habituative transmitter depend only on the self-excitatory feedback signal (see Equation 1.7) in the equation governing membrane potential dynamics (Equation 1.5), and not also on bottom-up excitatory inputs (see Equation 2.3). This gating is sufficient to prevent persistent firing of map cells that become intensely active and thereby allow other cells to participate in activity-dependent plasticity. Case 6 simulation results presented in Figures 13A and 13B (green curves) show that grid cell spatial scale gradient can be learned even when the habituative gating operates on both the weighted stripe cell inputs and the recurrent on-center feedback (see Equation 2.1). This is in part due to model robustness, and in part due to the relatively weaker driving force of bottom-up inputs compared to the self-excitatory feedback signal (see Figures 3B and 3C).

Fourth, the adaptive weights from stripe cells to category cells use a different version of the instar learning law (Equation 1.6) that more robustly enabled category cells to become tuned to coactivations of stripe cells [20]. The instar learning law used in GRIDSmap (Equation 2.2) could sometimes allow a category cell to get tuned to just one strong or sustained input neuron when its adaptive weight exhausts the weights available for learning in the other stripe cell pathways via term 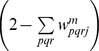 in Equation 2.2. As a result, stripe-like, rather than hexagonal, firing fields of grid cells could arise in two situations: more correlated activations of stripe cells when stripe cells exist with smaller separations between stripe directions, or more sustained activations of stripe cells with larger stripe fields (see Figures 8 and 9 in [23]). Instead, the current learning law allows each weight to track the ratio of stripe cell activities, time-averaged during intervals when the learning gate is open.

In the GRIDSmap model, the stripe cells of different spacings were assumed to have the same maximal firing rate but different field widths (i.e., 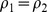 and 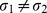 in Equation 1.4). In other words, the total firing in a stripe field was different across scales, so that the stripe cell receptive fields are not normalized. In contrast [6], reported that the peak firing rates of grid cells decrease from the dorsal to the ventral end of MEC while grid field widths increase, and Spectral Spacing model simulations show that normalized stripe cell receptive fields are needed to simulate all the data about how spatial and temporal properties of grid cell firing changes along the dorsoventral axis.

### Other grid cell models

Several models exist to explain the generation of grid cells, but the Spectral Spacing model differentiates itself by providing for the first time a principled explanation of how grid cells learn not only their characteristic hexagonal grid firing patterns [5], [37], [38] but also their spatial scale gradient along the dorsoventral axis of MEC [4], [6], and how this self-organization process relates to intrinsic cellular properties along the same axis [19]. These contributions represent significant breakthroughs, especially considering that few prior works address aspects of how grid cells may be learned in a self-organized manner [20], [23], [99].

Prior grid cell models can be generally classified into two categories based on whether the linear velocity path integration happens before or at the level of grid cells. In addition to the SOM type of model, the former possibility has been modeled using mechanisms of oscillatory interference [21], [22], [100] and ring (1-D periodic) attractors [36], [99]. In the family of models based on oscillatory interference, the inputs to grid cells at which path integration occurs have been called *band cells* [21]. Although band cells use different mechanisms than the stripe cells of SOM models [23], they also generate 1-D periodic spatial firing patterns (see Figure 1A). Models which implement path integration at the level of grid cells include toroidal (2-D periodic) attractor networks [8], [9], [101].

Oscillatory interference models [21], [22], [100] propose that the grid cell firing pattern forms from interference between membrane potential oscillations in different compartments within a single cell. These compartments include the cell soma, whose oscillation has a baseline theta frequency, and various dendritic compartments, whose oscillation frequencies are sensitive to linear velocity and head direction. In this way, displacement information can be implicitly encoded in the phase differences between the baseline oscillation and the different active oscillations. The dendritic oscillations are controlled by input band cells, which exhibit periodic firing with frequencies proportional to the linear velocity component along their preferred directions. The interference models assume each grid cell receives inputs from exactly three band cells whose preferred directions are 60° apart from each other in order to generate hexagonal grid spatial firing fields. Grid firing patterns different from hexagonal patterns, and which are not observed *in vivo*, result if this constraint is not met [22]. The interference models assume that the right input combination of band cells is selected through some self-organization process [21], but this has not yet been demonstrated. The existence of subthreshold oscillations in dMEC layer II stellate cells [12], [53] and their dorsoventral gradient [13], [14] are interpreted as strong evidence for an oscillatory interference-based mechanism for grid cells [13], [21], [22]. However, the Spectral Spacing model not only learns grid cells of multiple spatial scales without invoking oscillatory interference, but also accounts for their MPOs and, in particular, the gradient in oscillation frequencies along the dorsoventral axis of MEC as an epiphenomenon.

The 2-D attractor models [8], [9], [101] propose that grid cell properties result from network-level dynamics in a two-dimensional sheet of neurons. In the absence of any translational movement, persistent localized firing of grid cells is ensured by a recurrent on-center off-surround connectivity with symmetric weights between the cells. However in response to non-zero linear velocity signals, the connections among cells are activated in a directionally asymmetric manner to cause the activity pattern, or bump, to shift accordingly for the direct encoding of displacement information. 2-D spatially periodic firing fields arise from toroidal boundary conditions. While these models do not require an additional stage for the purpose of linear velocity path integration, it has not been demonstrated how a non-topographic periodic 2-D attractor network can be self-organized in the brain. A previous proposal for entraining such a network by a topographic aperiodic 2-D attractor network [9] has been suggested to be not feasible [101]. Moreover, 2-D attractor models have not yet provided a functional role for the gradient in the rate of temporal integration along the dorsoventral axis of MEC layer II [19]. They also do not yet account for the gradient in the frequency of subthreshold MPOs that are elicited in response to steady current injections [13], [14], and in the peak and mean firing rates [6]. While how a stripe cell ring attractor network self-organizes has also not yet been shown, it should be noted that [35] have shown how learning can adaptively calibrate vestibular, visual, and motor inputs to ring attractors that code head direction.

## References

[1] Dowding JE, Murphy EC (1994) Ecology of ship rats (*Rattus rattus*) in a Kauri (*Agathis australis*) forest in Northland, New Zealand. NZ J Ecol 18: 19–28.

[2] van Strien NM, Cappaert NLM, Witter MP (2009) The anatomy of memory: an interactive overview of the parahippocampal-hippocampal network. Nat Rev Neurosci 10: 272–282. 19300446 10.1038/nrn2614

[3] Fyhn M, Molden S, Witter MP, Moser EI, et al (2004) Spatial representation in the entorhinal cortex. Science 305: 1258–1264. 15333832 10.1126/science.1099901

[4] Sargolini F, Fyhn M, Hafting T, McNaughton B, Witter M, et al (2006) Conjunctive representation of position, direction, and velocity in entorhinal cortex. Science 321: 58–762. 10.1126/science.1125572 16675704

[5] Hafting T, Fyhn M, Molden S, Moser MB, Moser E (2005) Microstructure of the spatial map in the entorhinal cortex. Nature 436: 801–806. 15965463 10.1038/nature03721

[6] Brun VH, Solstad T, Kjelstrup KB, Fyhn M, Witter MP, et al (2008) Progressive increase in grid scale from dorsal to ventral medial entorhinal cortex. Hippocampus 18: 1200–1212. 19021257 10.1002/hipo.20504

[7] O'Keefe J, Burgess N (2005) Dual phase and rate coding in hippocampal place cells: Theoretical significance and relationship to entorhinal grid cells. Hippocampus 15: 853–866. 16145693 10.1002/hipo.20115 PMC2677681

[8] Fuhs MC, Touretzky DS (2006) A spin glass model of path integration in rat medial entorhinal cortex. J Neurosci 26: 4266–4276. 16624947 10.1523/JNEUROSCI.4353-05.2006 PMC6674007

[9] McNaughton BL, Battaglia FP, Jensen O, Moser EI, Moser MB (2006) Path integration and the neural basis of the “cognitive map”. Nat Rev Neurosci 7: 663–678. 16858394 10.1038/nrn1932

[10] Rolls ET, Stringer SM, Elliot T (2006) Entorhinal cortex grid cells can map to hippocampal place cells by competitive learning. Network 17: 447–465. 17162463 10.1080/09548980601064846

[11] Gorchetchnikov A, Grossberg S (2007) Space, time, and learning in the hippocampus: How fine spatial and temporal scales are expanded into population codes for behavioral control. Neural Net 20: 182–193. 10.1016/j.neunet.2006.11.007 17222533

[12] Alonso A, Klink R (1993) Differential electroresponsiveness of stellate and pyramidal-like cells of medial entorhinal cortex layer II. J Neurophysiol 70: 128–143. 8395571 10.1152/jn.1993.70.1.128

[13] Giocomo L, Zilli E, Fransen E, Hasselmo ME (2007) Temporal frequency of subthreshold oscillations scales with entorhinal grid cell field spacing. Science 315: 1719–1722. 17379810 10.1126/science.1139207 PMC2950607

[14] Yoshida M, Giocomo LM, Boardman I, Hasselmo ME (2011) Frequency of subthreshold oscillations at different membrane potential voltages in neurons at different anatomical positions on the dorsoventral axis in the rat medial entorhinal cortex. J Neurosci 31: 12683–12694. 21880929 10.1523/JNEUROSCI.1654-11.2011 PMC3177240

[15] Giocomo LM, Hasselmo ME (2008) Time constants of h current in layer II stellate cells differ along the dorsal to ventral axis of medial entorhinal cortex. J Neurosci 28: 9414–9425. 18799674 10.1523/JNEUROSCI.3196-08.2008 PMC2990529

[16] Giocomo LM, Hasselmo ME (2008) Computation by oscillations: Implications of experimental data for theoretical models of grid cells. Hippocampus 18: 1186–1199. 19021252 10.1002/hipo.20501 PMC2653064

[17] Nolan MF, Dudman JT, Dodson PD, Santoro B (2007) HCN1 channels control resting and active integrative properties of stellate cells from layer II of the entorhinal cortex. J Neurosci 27: 12440–12451. 18003822 10.1523/JNEUROSCI.2358-07.2007 PMC6673323

[18] Giocomo LM, Hasselmo ME (2009) Knock-out of HCN1 subunit flattens dorsal-ventral frequency gradient of medial entorhinal neurons in adult mice. J Neurosci 29: 7625–7630. 19515931 10.1523/JNEUROSCI.0609-09.2009 PMC2729850

[19] Garden DLF, Dodson PD, O'Donnell C, White MD, Nolan MF (2008) Tuning of synaptic integration in the medial entorhinal cortex to the organization of grid cell firing fields. Neuron 60: 875–889. 19081381 10.1016/j.neuron.2008.10.044

[20] Pilly PK, Grossberg S (2012) How do spatial learning and memory occur in the brain? Coordinated learning of entorhinal grid cells and hippocampal place cells. J Cogn Neurosci 24: 1031–1054. 22288394 10.1162/jocn_a_00200

[21] Burgess N, Barry C, O'Keefe J (2007) An oscillatory interference model of grid cell firing. Hippocampus 17: 801–812. 17598147 10.1002/hipo.20327 PMC2678278

[22] Hasselmo ME, Giocomo LM, Zilli EA (2007) Grid cell firing may arise from interference of theta frequency membrane potential oscillations in single neurons. Hippocampus 17: 1252–1271. 17924530 10.1002/hipo.20374 PMC2408670

[23] Mhatre H, Gorchetchnikov A, Grossberg S (2012) Grid cell hexagonal patterns formed by fast self-organized learning within entorhinal cortex. Hippocampus 22: 320–34. 21136517 10.1002/hipo.20901

[24] Krupic J, Burgess N, O'Keefe J (2011) Periodic bands are the building blocks of locational firing in the parahippocampal formation [Abstract 729.13]. In: Proceedings of the Annual Conference of the Society for Neuroscience; 12–16 November 2011; Washington, DC, United States. Neuroscience 2011. Available: http://www.abstractsonline.com/Plan/ViewAbstract.aspx?sKey=07c4898f-8c6a-43d0-b307-6374c69a19ac&cKey=ecdb7022-51ff-463c-a23b-1da7e29ffeaa&mKey=8334BE29-8911-4991-8C31-32B32DD5E6C8. Accessed 12 July 2012.

[25] Caballero-Bleda M, Witter MP (1993) Regional and laminar organization of projections from the presubiculum and parasubiculum to the entorhinal cortex: An anterograde tracing study in the rat. J Comp Neurol 328: 115–129. 8429124 10.1002/cne.903280109

[26] Caballero-Bleda M, Witter MP (1994) Projections from the presubiculum and the parasubiculum to morphologically characterized entorhial-hippocampal projection neurons in the rat. Exp Brain Res 101: 93–108. 7843307 10.1007/BF00243220

[27] Goldberg ME, Hudspeth AJ (2000) The vestibular system. In: Kandel ER, Schwartz JH, Jessell TM, editors. Principles of neural science, 4/e. New York: McGraw-Hill Press. pp. 801–815.

[28] Blair H, Sharp P (1995) Anticipatory head direction signals in anterior thalamus: evidence for a thalamocortical circuit that integrates angular head motion to compute head direction. J Neurosci 15: 6260–6270. 7666208 10.1523/JNEUROSCI.15-09-06260.1995 PMC6577663

[29] Skaggs WE, Knierim J, Kudrimoti HS, McNaughton BL (1995) A model of the neural basis of the rat's sense of direction. Adv Neur In 7: 173–180. 11539168

[30] Blair H, Sharp P (1996) Visual and vestibular influences on head direction cells in the anterior thalamus of the rat. Behav Neurosci 10: 643–660. 10.1037//0735-7044.110.4.643 8864258

[31] Redish AD, Elga AN, Touretzky DS (1996) A coupled attractor model of the rodent head direction system. Network-Comp Neural 7: 671–685.

[32] Goodridge JP, Touretzky DS (2000) Modeling attractor deformation in the rodent head-direction system. J Neurophysiol 83: 3402–3410. 10848558 10.1152/jn.2000.83.6.3402

[33] Boucheny C, Brunel N, Arleo A (2005) A continuous attractor network model without recurrent excitation: Maintenance and integration in the head direction cell system. J Comput Neurosci 18: 205–227. 15714270 10.1007/s10827-005-6559-y

[34] Song P, Wang XJ (2005) Angular path integration by moving “hill of activity”: A spiking neuron model without recurrent excitation of the head-direction system. J Neurosci 25: 1002–1014. 15673682 10.1523/JNEUROSCI.4172-04.2005 PMC6725619

[35] Fortenberry B, Gorchetchnikov A, Grossberg S (2012) Learned integration of visual, vestibular, and motor cues in multiple brain regions computes head direction during visually-guided navigation. Hippocampus 18 doi: 10.1002/hipo.22040. 10.1002/hipo.22040 22707350

[36] Blair HT, Gupta K, Zhang K (2008) Conversion of a phase- to a rate-coded position signal by a three-stage model of theta cells, grid cells, and place cells. Hippocampus 18: 1239–1255. 19021259 10.1002/hipo.20509 PMC2814603

[37] Langston RF, Ainge JA, Couey JJ, Canto CB, Bjerknes TL, et al (2010) Development of the spatial representation system in the rat. Science 328: 1576–1580. 20558721 10.1126/science.1188210

[38] Wills TJ, Cacucci F, Burgess N, O'Keefe J (2010) Development of the hippocampal cognitive map in preweanling rats. Science 328: 1573–1576. 20558720 10.1126/science.1188224 PMC3543985

[39] Grossberg S (1973) Contour enhancement, short-term memory, and constancies in reverberating neural networks. Stud Appl Math 52: 213–257.

[40] Grossberg S (1976) Adaptive pattern classification and universal recoding, I: Parallel development and coding of neural feature detectors. Biol Cybern 23: 121–134. 974165 10.1007/BF00344744

[41] Grossberg S, Seitz A (2003) Laminar development of receptive fields, maps, and columns in visual cortex: The coordinating role of the subplate. Cereb Cortex 13: 852–863. 12853372 10.1093/cercor/13.8.852

[42] Olson S, Grossberg S (1998) A neural network model for the development of simple and complex cell receptive fields within cortical maps of orientation and ocular dominance. Neural Netw 11: 189–208. 12662831 10.1016/s0893-6080(98)00003-3

[43] Grossberg S, Williamson JR (2001) A neural model of how horizontal and interlaminar connections of visual cortex develop into adult circuits that carry out perceptual grouping and learning. Cereb Cortex 11: 37–58. 11113034 10.1093/cercor/11.1.37

[44] Grossberg S (1980) How does the brain build a cognitive code? Psychol Rev 87: 1–51. 7375607 10.1007/978-94-009-7758-7_1

[45] Carpenter GA, Grossberg S (1987) A massively parallel architecture for a self-organizing neural pattern recognition machine. Comput Vis Graph Image Process 37: 54–115.

[46] Purves D (1988) Body and brain: a trophic theory of neural connections. Cambridge: Harvard University Press. 10.1126/science.244.4907.993 17731884

[47] Cabelli RJ, Hohn A, Shatz CJ (1995) Inhibition of ocular dominance column formation by infusion of NT-4/5 or BDNF. Science 267: 1662–1666. 7886458 10.1126/science.7886458

[48] Cabelli RJ, Shelton DL, Segal RA, Shatz CJ (1997) Blockade of endogenous ligands of trkB inhibits formation of ocular dominance columns. Neuron 19: 63–76. 9247264 10.1016/s0896-6273(00)80348-7

[49] Royer S, Pare D (2003) Conservation of total synaptic weight through balanced synaptic depression and potentiation. Nature 422: 518–522. 12673250 10.1038/nature01530

[50] Grossberg S, Schmajuk NA (1989) Neural dynamics of adaptive timing and temporal discrimination during associative learning. Neural Netw 2: 79–102.

[51] Grossberg S, Merrill JWL (1992) A neural network model of adaptively timed reinforcement learning and hippocampal dynamics. Cognitive Brain Res 1: 3–38. 10.1016/0926-6410(92)90003-a 15497433

[52] Grossberg S, Merrill JWL (1996) The hippocampus and cerebellum in adaptively timed learning, recognition, and movement. J Cogn Neurosci 8: 257–277. 23968151 10.1162/jocn.1996.8.3.257

[53] Klink R, Alonso A (1997) Muscarinic modulation of the oscillatory and repetitive firing properties of entorhinal cortex layer II neurons. J Neurophysiol 77: 1813–1828. 9114238 10.1152/jn.1997.77.4.1813

[54] Shalinsky MH, Magistretti J, Ma L, Alonso AA (2002) Muscarinic activation of a cation current and associated current noise in entorhinal-cortex layer-II neurons. J Neurophysiol 88: 1197–1211. 12205141 10.1152/jn.2002.88.3.1197

[55] Woodhall GL, Bailey SJ, Thompson SE, Evans DI, Jones RS (2005) Fundamental differences in spontaneous synaptic inhibition between deep and superficial layers of the rat entorhinal cortex. Hippocmapus 15: 232–245. 10.1002/hipo.20047 15386594

[56] Khwaja FA, Alonso AA, Bourque CW (2007) Ca(2+)-dependent K(+) currents and spike-frequency adaptation in medial entorhinal cortex layer II stellate cells. Hippocampus 17: 1143–1148. 17880008 10.1002/hipo.20365

[57] Tsanov M, Manahan-Vaughan D (2007) Intrinsic, light-independent and visual activity-dependent mechanisms cooperate in the shaping of the field response in rat visual cortex. J Neurosci 27: 8422–8429. 17670989 10.1523/JNEUROSCI.1180-07.2007 PMC6673071

[58] Gaudiano P, Grossberg S (1991) Vector associative maps: Unsupervised real-time error-based learning and control of movement trajectories. Neural Netw 4: 147–183.

[59] Izhikevich EM (2007) Dynamical systems in neuroscience: The geometry of excitability and bursting. Cambridge: MIT Press.

[60] Dickson CT, Magistretti J, Shalinsky MH, Fransen E, Hasselmo ME, et al (2000) Properties and role of I(h) in the pacing of subthreshold oscillations in entorhinal cortex layer II neurons. J Neurophysiol 83: 2562–2579. 10805658 10.1152/jn.2000.83.5.2562

[61] Lancaster B, Adams PR (1986) Calcium-dependent current generating the afterhyperpolarization of hippocampal neurons. J Neurophysiol 55: 1268–1282. 2426421 10.1152/jn.1986.55.6.1268

[62] Giocomo LM, Hussaini SA, Zheng F, Kandel ER, et al (2011) Grid cells use HCN1 channels for spatial scaling. Cell 147: 1159–1170. 22100643 10.1016/j.cell.2011.08.051

[63] Barry C, Hayman R, Burgess N, Jeffery K (2007) Experience-dependent rescaling of entorhinal grids. Nat Neurosci 10: 682–684. 17486102 10.1038/nn1905

[64] Grossberg S (1978) A theory of human memory: Self-organization and performance of sensory-motor codes, maps, and plans. In: Rosen R, Snell F, editors. Progress in theoretical biology, Volume 5. New York: Academic Press. pp. 233–374.

[65] Chey J, Grossberg S, Mingolla E (1998) Neural dynamics of motion processing and speed discrimination. Vision Res 38: 2769–2786. 9775325 10.1016/s0042-6989(97)00372-6

[66] Grossberg S (1994) 3-D vision and figure-ground separation by visual cortex. Percept Psychophys 55: 48–120. 8036093 10.3758/bf03206880

[67] Grossberg S, Myers CW (2000) The resonant dynamics of speech perception: Interword integration and duration-dependent backward effects. Psychol Rev 107: 735–767. 11089405 10.1037/0033-295x.107.4.735

[68] Grossberg S, Kazerounian S (2011) Laminar cortical dynamics of conscious speech perception: A neural model of phonemic restoration using subsequent context in noise. J Acoust Soc Am 130: 440–460. 21786911 10.1121/1.3589258

[69] Dodson PD, Pastoll H, Nolan MF (2011) Dorsal-ventral organization of theta-like activity intrinsic to entorhinal stellate neurons is mediated by differences in stochastic current fluctuations. J Physiol 589: 2993–3008. 21502290 10.1113/jphysiol.2011.205021 PMC3139082

[70] Dolorfo CL, Amaral DG (1998) Entorhinal cortex of the rat: Topographic organization of the cells of origin of the perforant path projection to the dentate gyrus. J Comp Neurol 398: 25–48. 9703026

[71] Jeewajee A, Barry C, O'Keefe J, Burgess N (2008) Grid cells and theta as oscillatory interference: electophysiological data from freely moving rats. Hippocampus 18: 1175–1185. 19021251 10.1002/hipo.20510 PMC3173868

[72] Boehlen A, Heinemann U, Erchova I (2010) The range of intrinsic frequencies represented by medial entorhinal cortex stellate cells extends with age. J Neurosci 30: 4585–4589. 20357109 10.1523/JNEUROSCI.4939-09.2010 PMC6632313

[73] Bonnevie T, Fyhn M, Hafting T, Derdikman D, Moser EI, et al (2010) Hippocampal contribution to maintenance of entorhinal grid fields [Abstract 101.4]. In: Proceedings of the Annual Conference of the Society for Neuroscience; 13–17 November 2010; San Diego, CA, United States. Neuroscience 2010. Available: http://www.abstractsonline.com/Plan/ViewAbstract.aspx?sKey=1cdafe8e-ffef-47d1-b94d-72f2a6793390&cKey=a4f1eb94-d3d6-4291-97ce-3a41cc5e99b7&mKey=E5D5C83F-CE2D-4D71-9DD6-FC7231E090FB. Accessed 12 July 2012.

[74] Thompson LT, Best PJ (1990) Long-term stability of the place-field activity of single units recorded from the dorsal hippocampus of freely behaving rats. Brain Res 509: 299–308. 2322825 10.1016/0006-8993(90)90555-p

[75] Wilson MA, McNaughton BL (1993) Dynamics of the hippocampal ensemble code for space. Science 261: 1055–1058. 8351520 10.1126/science.8351520

[76] Muller RA (1996) A quarter of a century of place cells. Neuron 17: 813–822. 8938115 10.1016/s0896-6273(00)80214-7

[77] Frank LM, Stanley GB, Brown EN (2004) Hippocampal plasticity across multiple days of exposure to novel environments. J Neurosci 24: 7681–7689. 15342735 10.1523/JNEUROSCI.1958-04.2004 PMC6729632

[78] Grossberg S (1999) The link between brain learning, attention, and consciousness. Conscious Cogn 8: 1–44. 10072692 10.1006/ccog.1998.0372

[79] Grossberg S (1976) Adaptive pattern classification and universal recoding, II: Feedback, expectation, olfaction, and illusions. Biol Cybern 23: 187–202. 963125 10.1007/BF00340335

[80] Grossberg S (2009) Beta oscillations and hippocampal place cell learning during exploration of novel environments. Hippocampus 19: 881–885. 19370545 10.1002/hipo.20602

[81] Kentros CG, Agniotri NT, Streater S, Hawkins RD, Kandel ER (2004) Increased attention to spatial context increases both place field stability and spatial memory. Neuron 42: 283–295. 15091343 10.1016/s0896-6273(04)00192-8

[82] Kentros CG, Hargreaves E, Hawkins RD, Kandel ER, Shapiro M, et al (1998) Abolition of long-term stability of new hippocampal place cell maps by NMDA receptor blockade. Science 280: 2121–2126. 9641919 10.1126/science.280.5372.2121

[83] Morris RGM, Frey U (1997) Hippocampal synaptic plasticity: role in spatial learning or the automatic recording of attended experience? Philos T Roy Soc B 1360: 1469–1503. 10.1098/rstb.1997.0136 PMC1692060 9368938

[84] Fransen E, Alonso AA, Dickson CT, Magistretti J, Hasselmo ME (2004) Ionic mechanisms in the generation of subthreshold oscillations and action potential clustering in entorhinal layer II stellate neurons. Hippocampus 14: 368–384. 15132436 10.1002/hipo.10198

[85] Schreiber S, Erchova I, Heinemann U, Herz AV (2004) Subthreshold resonance explains the frequency-dependent integration of periodic as well as random stimuli in the entorhinal cortex. J Neurophysiol 92: 408–415. 15014100 10.1152/jn.01116.2003

[86] Alonso A, Llinas RR (1989) Subthreshold Na^+^-dependent theta-like rhythmicity in stellate cells of entorhinal cortex layer II. Nature 342: 175–177. 2812013 10.1038/342175a0

[87] Navratilova Z, Giocomo LM, Fellous JM, Hasselmo ME, McNaughton BL (2012) Phase precession and variable spatial scaling in a periodic attractor map model of medial entorhinal grid cells with realistic after-spike dynamics. Hippocampus 22: 772–789. 21484936 10.1002/hipo.20939

[88] MacDonald CJ, Lepage LQ, Eden UT, Eichenbaum H (2011) Hippocampal “time cells” bridge the gap in memory for discontiguous events. Neuron 71: 737–749. 21867888 10.1016/j.neuron.2011.07.012 PMC3163062

[89] Fiala JC, Grossberg S, Bullock D (1996) Metabotropic glutamate receptor activation in cerebellar Purkinje cells as substrate for adaptive timing of the classically conditioned eye blink response. J Neurosci 16: 3760–3774. 8642419 10.1523/JNEUROSCI.16-11-03760.1996 PMC6578825

[90] Brown J, Bullock D, Grossberg S (1999) How the basal ganglia use parallel excitatory and inhibitory learning pathways to selectively respond to unexpected rewarding cues. J Neurosci 19: 10502–10511. 10575046 10.1523/JNEUROSCI.19-23-10502.1999 PMC6782432

[91] Hargreaves EL, Rao G, Lee I, Knierim JJ (2005) Major dissociation between medial and lateral entorhinal input to dorsal hippocampus. Science 308: 1792–1794. 15961670 10.1126/science.1110449

[92] Aminoff E, Gronau N, Bar M (2007) The parahippocampal cortex mediates spatial and nonspatial associations. Cereb Cortex 17: 1493–1503. 16990438 10.1093/cercor/bhl078

[93] Kerr KM, Agster KL, Furtak SC, Burwell RD (2007) Functional neuroanatomy of the parahippocampal region: the lateral and medial entorhinal areas. Hippocampus 17: 697–708. 17607757 10.1002/hipo.20315

[94] Eichenbaum H, Lipton PA (2008) Towards a functional organization of the medial temporal lobe memory system: Role of the parahippocampal and medial entorhinal cortical areas. Hippocampus 18: 1314–1324. 19021265 10.1002/hipo.20500 PMC2592493

[95] Tulving E (1972) Episodic and semantic memory. In: Tulving E, Donaldson W, editors. Organization of Memory. New York: Academic Press.

[96] Tulving E, Thomson DC (1973) Encoding specificity and retrieval processes in episodic memory. Psychol Rev 80: 352–373.

[97] Tulving E (1984) Précis of elements of episodic memory. Behav Brain Sci 7: 223–268.

[98] Eichenbaum H, Dudchenko P, Wood E, Shapiro M, Tanila H (1999) The hippocampus, memory and place cells: Is it spatial memory or a memory space? Neuron 23: 209–226. 10399928 10.1016/s0896-6273(00)80773-4

[99] Gaussier P, Banquet JP, Sargolini F, Giovannangeli C, Save E, et al (2007) A model of grid cells involving extra-hippocampal path integration, and the hippocampal loop. J Integr Neurosci 6: 447–476. 17933021 10.1142/s021963520700160x

[100] Burgess N (2008) Grid cells and theta as oscillatory interference: theory and predictions. Hippocmapus 18: 1157–1174. 10.1002/hipo.20518 PMC3196519 19021256

[101] Burak Y, Fiete IR (2009) Accurate path integration in continuous attractor network model of grid cells. PLoS Comput Biol 5: e1000291. 19229307 10.1371/journal.pcbi.1000291 PMC2632741

